# Tailoring hyaluronic acid hydrogels: Impact of cross-linker length and density on skin rejuvenation as injectable dermal fillers and their potential effects on the MAPK signaling pathway suppression

**DOI:** 10.1016/j.bioactmat.2025.03.002

**Published:** 2025-03-08

**Authors:** Mohanapriya Murugesan, Ramya Mathiyalagan, Zelika Mega Ramadhania, Jinnatun Nahar, Cuong Hung Luu, V.H. Giang Phan, Deok Chun Yang, Qihui Zhou, Se Chan Kang, Thavasyappan Thambi

**Affiliations:** a Graduate School of Biotechnology, College of Life Sciences, Kyung Hee University, Yongin Si, Gyeonggi do, 17104, Republic of Korea; b Queensland Micro- and Nanotechnology Centre, Griffith University, Nathan, QLD, 4111, Australia; c Biomaterials and Nanotechnology Research Group, Faculty of Applied Sciences, Ton Duc Thang University, Ho Chi Minh City, Vietnam; d Department of Oriental Medicinal Biotechnology, College of Life Science, Kyung Hee University, Yongin-si, Gyeonggi-do, 17104, Republic of Korea; e Qingdao Key Laboratory of Materials for Tissue Repair and Rehabilitation, School of Rehabilitation Sciences and Engineering, University of Health and Rehabilitation Sciences, Qingdao, 266071, China

**Keywords:** Hyaluronic acid, Cross-linking, Injectability, Dermal filler, Anti-wrinkling, MAPK signaling pathway, Collagen deposition

## Abstract

Hyaluronic acid (HA) hydrogels, obtained through cross-linking, provide a stable 3D environment that is important for controlled delivery and tissue engineering applications. Cross-linking density has a significant impact on the physicochemical properties of hydrogels, including their shape stability, mechanical stiffness and macromolecular diffusivity. However, often cross-linking chemistries require photoinitiator and catalyst that may be toxic and cause unwanted tissue response. Here, we prepared a series of HA hydrogel with varying cross-linker length and cross-linking density, which can be obtained by altering the feed ratio of three different cross-linkers from small molecules to macromolecules (e.g., 1,4-butanediol diglycidyl ether (BDDE), ferulic acid (FA), pluronic (PLU)), to ameliorate skin wrinkles in mice models. HA cross-linked with FA and PLU exhibited enzyme and temperature-dependent sol-to-gel phase transition, respectively, and the gels possess good injectability. In vitro test confirmed that HA hydrogels co-cultured with RAW 264.7 and HDF cells showed good biocompatibility. In particular, HA cross-linked with PLU stimulated the growth of HDF cells and HaCaT cells. HA cross-linked with PLU suppressed the expression levels of proteins involved in collagen degradation including mitogen-activated protein kinases (ERK, JNK, p38) and matrix metalloproteases (MMP-1, MMP-3, and MMP-9) resulting in increased deposition of Collagen I. The free-flowing sols of HA hydrogel precursors are subcutaneously injected into the back of BALB/c mice and form stable gels at the dermis layer and found to be non-toxic. More importantly, HA hydrogel cross-linked with PLU showed an enhanced anti-wrinkling effect in the wrinkled mice model. Thus, properties of HA hydrogels such as injectability, biocompatibility, and good anti-wrinkling effect altered through varying cross-linking density must be considered in the context of soft tissue engineering applications.

## Highlights

• Hyaluronic acid hydrogels with varied cross-linker lengths and cross-linking densities were synthesized without any catalyst.
• The hyaluronic acid hydrogel shows good injectability, printability and enhanced mechanical properties.
• The biocompatible hyaluronic acid hydrogel promotes cell proliferation and migration of dermal fibroblast and human keratinocyte cells.
• The dynamic hydrogel can effectively suppress MAPK/AP-1 (JNK/c-Jun) signaling pathway and increase the deposition of Collagen I.
• Subcutaneously injected hyaluronic acid hydrogel effected induced the anti-wrinkling effect in mice models by restoration of collagen.

## Graphical abstract

**Figure:**
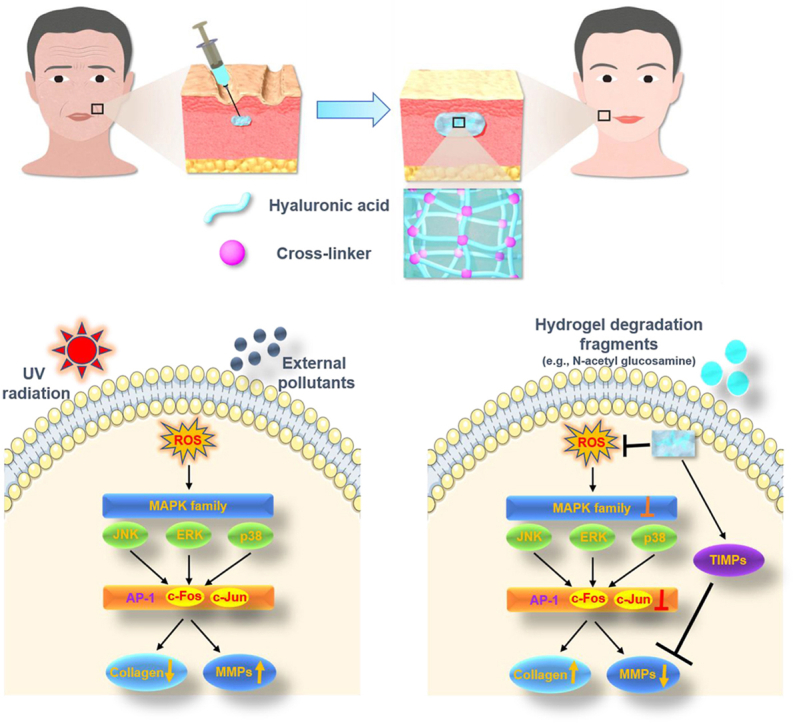

## 1 Introduction

Hydrogels are three-dimensional (3D) polymeric networks that are hydrophilic or amphiphilic. They possess unique properties, including the ability to mimic the extracellular matrix (ECM) of tissues, support cell growth, control the release of therapeutic agents, and minimize invasiveness and mechanical irritation when injected into surrounding tissues [[1], [2], [3], [4], [5], [6]], and are therefore directly applied in cosmetics etc. [7,8]. Particularly, injectable hydrogels are promising biomaterials for tissue engineering applications since they can be injected into the deep tissues by using a catheter and also have the shape-filling ability due to their moldability and high hydration properties with minimal invasiveness [[9], [10], [11], [12], [13]]. The injectable and malleable properties of hydrogels allow them to fill in 3D spaces [14,15], which could broaden their applications from local delivery to regenerative medicine [[16], [17], [18]].

Hyaluronic acid (HA) is a naturally abundant polysaccharide made up of repeating disaccharide units consisting of D-glucuronic acid and *N*-acetyl D-glucosamine linked via β1→3 and β1→4 glycosidic linkages [19,20]. HA is an abundant linear polysaccharide found in connective tissues and other major organs, and is available in a range of molecular weights [[21], [22], [23]]. HA exhibits excellent biocompatibility, biodegradability, non-immunogenic and viscoelastic properties and therefore has been used to prepare biomaterials for various biomedical applications [24,25]. Because of the materials abundance, affordability, and high reproducibility of the developed hydrogels make the HA as an ideal candidate to prepare biomaterials [26,27]. In particular, HA confers a smooth texture and softness to the skin [28] and therefore HA-based biomaterials are often injected or topically applied to prevent dehydration, maintain elasticity, and combat aging and wrinkles [29]. In particular, HA-based injectable fillers are often used to treat anti-wrinkling applications.

Mechanically robust HA-based biomaterials have been synthesized using a variety of chemical modifications [[30], [31], [32]]. These HA derivatives exhibit improved physicochemical properties without compromising the biocompatible and biodegradable nature of native HA [[33], [34], [35], [36]]. Often, the simplest method used to prepare HA hydrogel is cross-linking [36,37]. Nevertheless, the resilience of HA hydrogels relies on their stability against degradation by hyaluronidases and reactive oxygen species (ROS), which in turn limits their biomedical applications [38]. To surmount the issues associated with HA degradation, functional groups of HA are often utilized in the preparation of HA-based biomaterials [39,40]. Often, the most commonly used sites of covalent modification in HA are hydroxyl group, carboxylic group, and –NH–CO–CH_3_ group [41]. Hydroxyl group of HA can be easily modified into four different derivatives of HA such as ether, ester, hemiacetal or oxidation [42]. However, most of the cross-linking strategies in HA functionalization employs multistep synthesis and often utilizes cytotoxic photoinitiators and catalysts. Therefore, simple cross-linking strategies required to obtain HA-based hydrogels fillers that can not only reduce the complexity of the existing system and also improve the reproducibility of the HA-based hydrogel fillers. Such formulations can reduce the risk of skin inflammation and other adverse events at the site of injection, which lead to the generation of inflamed skin and subsequently inhibit the regeneration of collagen.

Skin acts as a barrier between organism and environment, effectively protects the body from external stimuli such as heat, visible light, and ultraviolet (UV) exposure [43]. Long-term exposure to UV radiation is a major causative factor in skin aging, leading to wrinkle formation, dryness, pigmentation, and inflammation [44]. This is mainly due to the cellular stress and production of ROS on human skin [45]. Among different UV radiation, UVB radiation (280–320 nm) regarded as “the burning rays” can promote the generation of intracellular ROS, which in turn generate inflammatory mediators and induce collagen degradation [46,47]. Therefore, UVB irradiation on skin tissues not only inhibit collagen synthesis but also facilitates collagenolysis by upregulating matrix metalloproteases (MMPs) expression [48]. UVB-induced ROS activates mitogen-activated protein kinase (MAPK) signaling, which triggers the secretion of MMPs, leading to collagen degradation [49]. Therefore, it is important to develop hydrogel fillers that suppress the MAPK/AP-1 (JNK/c-Jun) signaling pathway by impeding gene transcription of MMPs and promoting collagen regeneration.

In this study, we demonstrate the preparation of cross-linked HA hydrogels with three different strategies, by using three different cross-linkers, to improve physiological, rheological and safety of cross-linked HA biomaterials used in cosmetic applications, specifically anti-wrinkling treatments. The length and density of cross-linkers significantly influence the physicochemical properties of HA hydrogels, such as mechanical strength, injectability, and biodegradability. These attributes are critical for skin-related applications, including tissue regeneration and anti-wrinkle therapies, where stability and biocompatibility are paramount. Optimizing cross-linker characteristics enables the development of HA hydrogels with tailored properties, enhancing their therapeutic efficacy and integration into target tissues. To achieve this, we selected three cross-linkers used in this study, 1,4-butanediol diglycidyl ether (BDDE), ferulic acid (FA), and pluronic (PLU), based on their distinct structural attributes and contributions to hydrogel functionality. These structural variations directly influence the hydrogels’ physicochemical properties, including mechanical strength, biodegradability, and responsiveness, aligning with the requirements of tissue engineering and anti-wrinkle therapies. The prepared cross-linked HA biomaterials are subjected to various analysis, including, morphology, mechanical properties, hemocompatibility, and biocompatibility. The proliferation of human dermal fibroblast (HDF) cells incubated with various HA-based hydrogels have been investigated using Live/Dead imaging. The suppression of MAPK/AP-1 (JNK/c-Jun) signaling pathway was investigated using reverse transcription polymerase chain reaction (RT-PCR) analysis. Cell migration assay was carried out in HaCaT cells to examine the wound healing property of the HA-based hydrogels. Furthermore, in vivo gel formation of the HA-based hydrogels was investigated by implanting the gel precursors into the dermis layers of BALB/c mice. Finally, the in vivo application of HA-based hydrogels is examined in wrinkle mice model and its properties as injectable dermal fillers was confirmed by quantifying the amount of collagen deposition.

## 2 Materials and methods

### 2.1 Materials

Pluronic F127 (PLU), FA, carbonyl diimidazole (CDI), and BDDE were bought from Sigma-Aldrich (St. Louis, MO). Cell culture reagents, including phosphate-buffered saline (PBS), ethlyenediaminetetraacetic acid (EDTA), trypsin-EDTA, 4′,6-diamidino-2-phynylindole (DAPI), 6 normal solution of hydrochloric acid (6N HCl), Dulbecco's Modified Eagle's medium (DMEM), RPMI 1640 medium, fetal bovine serum (FBS) and penicillin-streptomycin, were obtained from Invitrogen (Carlsbad, CA).

### 2.2 Synthesis of BDDE cross-linked HA (HAB)

**Scheme 1:**
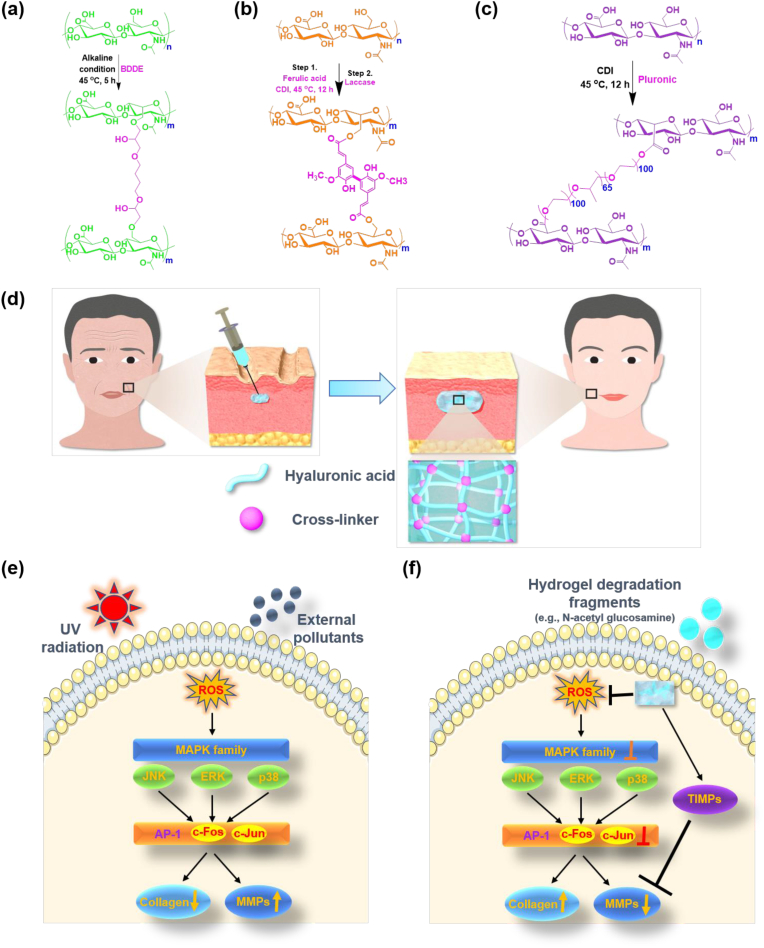
Schematic illustration of the preparation of HA-based hydrogel and its use as soft tissue fillers in anti-wrinkling applications. (a–c) Synthesis of HA based cross-linked polymers ((a) HAB, (b) HAF, and (c) HAP). (d) Injection of HA-based hydrogels into the wrinkle model promotes collagen deposition and induce anti-wrinkling effect. (e and f) The injection of HA based crosslinked polymers effectively inhibited the production of ROS and subsequently suppress the signaling of MAPK family proteins. This eventually reduce the degradation of MMPs and induce collagen deposition.

HA was dissolved in 10 mL of 1 % NaOH (wt./v). Thereafter, BDDE was slowly added to the HA solution and stirred vigorously. The feed molar ratio of BDDE to the hydroxyl group of HA was 0.05, 0.10, or 0.15. The reaction mixture was then allowed to cross-link at 45 °C for 5 h (Scheme 1a). PBS was then added to the above cross-linked HA, after which solution was transferred to dialysis bag and dialyzed sequentially against PBS and deionized water (DW) to remove the residual BDDE and NaOH.

### 2.3 Synthesis of FA cross-linked HA (HAF)

HAFs were synthesized by one-pot two-step process, as shown in Scheme 1b. In the first step, FA was activated using CDI [50]. Briefly, in a two-neck round-bottom flask equipped with a magnetic bar, equivalent amounts of FA and CDI (1.5 mmol) were dissolved in DMSO (20 mL). The reaction mixture was then stirred at 45 °C for 12 h. In the second step, varying amounts of HA was dissolved in formamide and slowly added to the CDI-activated FA and continued the stirring at the same temperature for an additional 12 h. At the end of reaction, the yellowish solution obtained was diluted in methanol and dialyzed against excess DW. Finally, HAF was lyophilized and stored at 4 °C.

### 2.4 Synthesis of PLU cross-linked HA (HAP)

HAPs were synthesized by one-pot two-step process, as shown in Scheme 1c. In the first step, HA was activated by CDI through esterification process. Briefly, 1 g of HA was dissolved in formamide followed by the addition of CDI and stirred for 6 h at 45 °C for 12 h. Thereafter, varying amount of PLU (feed molar ration 0.05, 0.10, or 0.15) dissolved in DMSO was added to the CDI-activated HA and stirred. After 12 h, the HAP mixtures were transferred to dialysis tube and dialyzed against excess water at 0 °C followed by lyophilization to obtain HAPs.

### 2.5 Characterization

***Nuclear magnetic resonance (NMR) spectra.*** The chemical structures of HAB, HAF and HAP were confirmed using ^1^H NMR spectra (Unity Inova 500WB NMR system) for the which the samples were dissolved in DMSO-*D*_6_ and *D*_2_O.

***Fourier Transform Infrared (FT-IR) spectra.*** FT-IR spectra of HAB, HAF and HAP were recorded on PerkinElmer Spectrum One system using KBr pellet methods.

***Scanning electron microscopy (SEM).*** Cross-sections of hydrogels were characterized by SEM. For SEM imaging, frozen hydrogels were freeze-dried for 48 h. Thereafter, thin slices of hydrogels mounted on a carbon tapes and coated with an ultrathin layer of gold. The microstructures of the lyophilized gels were observed by a LEO SUPRA 55, GENESIS 2000 (USA).

***Thermogravimetric analysis (TGA).*** TGA was performed under N_2_ atmosphere with a heating rate of 10 °C/min using TGA Q5000 IR/SDT Q600 (TA) (DE, USA).

### 2.6 Sol-to-gel phase transition

In a 4 mL flat-bottom vial, HAB, HAF, and HAP were separately dissolved in PBS (10 wt%) under vigorous stirring at 0 °C. The samples were placed in a water bath at 37 °C for 10 min and the physical state of the gel was observed by tilting the vials [51]. In case of HAF, laccase (10 U/mL) was added before incubation. The formulations were recognized as gels if not flowed after 2 min. On the other hand, if it starts flows it consider as sols.

The HA-based cross-linked gels formed in this study were transferred to 24- or 26-gauge needle (24G or 26G) hypodermic needles and examined their extrudability and shape moldability.

### 2.7 Swelling ratio

To evaluate the swelling ratio, the weight difference before and after soaking in PBS was measured. Initially, the sample was weighed in its dry state (W_0_), then immersed in PBS (pH 7.4) at 37 °C. At various time points (0, 10, 20, 30, 45, 60, 90, and 120 min), the samples were gently removed, blotted with filter paper to remove excess liquid, and their weights (W_t_) were recorded. The swelling ratio was then calculated using the following equation [52]:

Swelling ratio (%) = (W_t_ - W_0_)/ W_0_) × 100

### 2.8 Mechanical properties measurements

The compressive properties of the hydrogels were performed using a Universal Testing Machine (Instron, MA, USA). Compressive test on hydrogels were conducted on cylindrically molded samples (1.5 cm in diameter and 2.5 cm in height). The samples are placed it on the lower plate and compressed at a strain rate of 0.2 mm/s at the room temperature. The compressive modulus was calculated between 10 and 20 % strains [53].

### 2.9 Hemolysis test

The hemocompatibility of the hydrogels was determined using red blood cells (RBCs) [54]. To examine the hemocompatibility of hydrogels, blood from the mice was withdrawn by retro-orbital puncture and were stabilized with 5 μL of EDTA. The collected blood samples (1 mL) were mixed with PBS (2 mL) and centrifuged for 15 min at 1000 rpm. The supernatant was discarded and the RBCs were diluted with ice cold PBS (10 mL) and stored at 0 °C until further use. To determine hemocompatibility of hydrogels, 0.2 mL RBCs were added to 0.8 mL of various hydrogel formulations (20 mg/mL) and allowed to mix well. The RBCs mixed with equal volume of PBS and DW were considered as negative and positive control, respectively. Thereafter, the samples were incubated at 37 °C for 4 h and subsequently centrifuged for 10 min at 1500 rpm. The supernatants (100 μL) were transferred to 96-well plates and the absorbance at 541 nm was measured using microplate reader. Finally, the hemolytic activity of hydrogels was calculated using the formula as follows:

Hemolysis (%) = [(A_Sample_ – A_negative_)/ (A_positive_ – A_negative_)] × 100

### 2.10 In vitro biocompatibility test

***Cell culture.*** For the cell experiments, HDF cells, HaCaT cells, and RAW 264.7 cells were purchased from American Type Culture Collection (VA, USA). The cells were grown in DMEM or RPMI supplemented with 10 % FBS and 1 % penicillin-streptomycin in a humidified atmosphere at 37 °C.

***Biocompatibility test.*** To examine the biocompatibility of hydrogels, the cells (1 × 10^4^ cells/well) were co-cultured with different concentration of cross-linked HA formulations (varying from 100 to 1000 μg/mL). After 24 h, MTT solution (5 mg/mL) was added and incubated further for 3 h. The supernatant was then removed and purple formazan crystals were dissolved in DMSO. The absorbance of formazan was measured at 570 nm using microplate reader. The cell viability of the hydrogels was calculated by comparing the absorbance value of cells incubated with only culture medium.

***Live-dead cell imaging.*** HDF cells (1 × 10^4^ cells/well) were incubated with hydrogels (1000 μg/mL) at 37 °C in DMEM for various time intervals (e.g., 1 day, 3 days, 5 days). After the specified incubation periods, the cells were stained using a Live/Dead viability/cytotoxicity kit (Invitrogen, Carlsbad, CA) and visualized by a confocal laser scanning microscope (A1R; Nikon, Japan). The fluorescent images were processed using the Image J software to calculate the mean fluorescence intensity (MFI).

### 2.11 Scratch healing assay

For scratch healing assay, HaCaT cells were seeded in 12 well plates (2.5 × 10^5^ cells/well) and allowed to grow until reaching a confluent monolayer. The layers were scratched using 10 μL pipette tip to form a cell-free zone. After creating the cell-free zone, the wells were washed with PBS twice to remove the cellular debris formed during scratching. The cells were then treated with only culture medium (control) and hydrogel formulations (PLU and HAP) prepared in culture medium with the highest concentration used in biocompatibility test (1000 μg/mL). The cells were then allowed to proliferate for 48 h and images were captured using light microscope. The images were processed using the Image J software and the scratch healing or wound contraction percentage was calculated using the previously reported procedure [55].

### 2.12 Reverse transcription polymerase chain reaction (RT-PCR)

Hydrogels treated human keratinocytes (HaCaT cells) were digested using TRIzol reagent and the total RNA was isolated for the RT-PCR analysis. For RT-PCR analysis, an equal amount of RNA was reverse-transcribed into cDNA, and RT-PCR was carried out to evaluate the expression of various proteins. The primers used in this study are presented in Table 1. At the end of the RT-PCR, the product was electrophoresed using 1 % agarose gel and the bands in the gels were visualized with ethidium bromide staining. As an internal control, we used Glyceraldehyde-3-phosphate dehydrogenase (GAPDH).

**Table 1:** Primer sequences used for RT-PCR.

Genes	Primer sequences (5′-3′)	
	Forward	Reverse
ERK	5′-CCTAAGGAAAAGCTCAAAGA-3′	5′-AAAGTGGATAAGCCAAGAC-3′
JNK	5′-CGGTGAGGAACTACGTGGAG-3′	5′-ACCATCGCTCTCAACCCTTG-3′
p38	5′-CGACTTGCTGGAGAAGATGC-3′	5′-TCCATCTCTTCTTGGTCAAGG-3′
MMP-1	5′-ATTCTACTGATATCGGGGCTTTGA-3′	5′-ATGTCCTTGGGGTATCCGTGTAG-3′
MMP-3	5′-CTTTTGATGGACCTGGAAAAGT-3′	5′-GAGTGATAGAGACCCAGGGAAT-3′
MMP-9	5′-CGTCGTGATCCCCACTTACT-3′	5′-AGAGTACTGCTTGCCCAGGA-3′
Collagen I	5′-TGACGAGACCAAGAACTG-3′	5′-TACCAGGGTTTGAGCTCAGC-3′
GAPDH	5′-TGGACCTGACCTGCCGTCTA-3′	5′-GGCCTAAGGTCCACTTGTGTCA-3′
IL-6	5′-CCGGAGAGGAGACTTCACAG	5′-GGAAATTGGGGTAGGAAGGA-3′
TNF-α	5′-GCCAGAATGCTGCAGGACTT-3′	5′-GGCCTAAGGTCCACTTGTGTCA-3′

### 2.13 Western blot analysis

HDF cells were seeded into 6-well plates at a density of 1 × 10^6^ cells/well and treated with hydrogel formulations. Following the hydrogel treatment, cells were washed with ice-cold PBS and lysed for 30 min using radioimmunoprecipitation assay lysis buffer. The cell suspension was then centrifuged for 5 min at 12,000 rpm at 4 °C. Subsequently, the protein concentration in the supernatant was determined using the Bradford protein assay. For Western blotting, equal amounts of protein were separated by 10 % sodium dodecyl sulfate-polyacrylamide gel electrophoresis (SDS-PAGE) and blocked with 5 % skim milk in Tris-buffered saline with Tween-20. The proteins were transferred onto PVDF membranes and probed with primary antibodies against MMP-3. Bound antibodies were then detected with a horseradish peroxidase-linked secondary antibody (Cell Signaling Technology Inc., Danvars, MA, USA). Finally, proteins signals were visualized using the LAS-3000 Bio Imaging System (Fuji Film Co., Tokyo, Japan).

### 2.14 Gel formation in vivo and biodegradation

For in vivo gel formation, male BALB/c mice, aged 6–8 weeks (20–25 g), were used in all experiments to maintain consistency and avoid potential gender-based variability in results. The mice were housed in temperature- and humidity-controlled environments with a 12-h light/dark cycle and had ad libitum access to food and water.

For in vivo injection, HAB, HAF or HA hydrogels (10 wt%), PLU (10 wt%) and PLU (20 wt%) hydrogel precursors were prepared in PBS at 0 °C. Specifically, hydrogel precursors are dispersed in PBS and vigorously stirred at 0 °C in an ice bath. The free-flowing sols (200 μL) are then transferred to a 1 mL hypodermic syringe equipped with a 24G and kept in the ice bath for 15 min to prevent premature gelation. Following this, the sols are injected into the dorsal region of BALB/c mice that had been anaesthetized prior to the injection, where the body temperature facilitates the formation of the HAP hydrogel. To mitigate potential effects on surrounding cells, the formulation is extruded smoothly through the 24G to minimize damage and irritation to the tissue. After 30 min, the mice were sacrificed and the implantation site was cut open and photographed to examine the gel formation.

To investigate the in vivo biodegradation properties of hydrogels, the HAP-0.05 hydrogel precursors were prepared at a concentration of 10 wt% in PBS at 0 °C. Subsequently, sols (200 μL) were carefully transferred into a 1 mL syringe equipped with a 24G and administered into the dorsal region of BALB/c mice following prior anesthesia. For the control samples, identical volumes (200 μL) of 10 wt% or 20 wt% of PLU precursors were utilized. At specific time points (1 day, 7 days, and 21 days), the mice were sacrificed, and the biodegradation of the hydrogels was assessed by carefully opening the implantation site. The remaining hydrogel weight was determined using the mass-loss method [56].

### 2.15 Anti-wrinkling efficacy of hydrogels in vivo

To investigate the anti-wrinkling efficacy of hydrogels, mice with wrinkles were prepared using the previously reported procedure [57]. To develop wrinkles in mice, mixture of acetone/ether (1:1) soaked in slices of cotton was applied onto the rostral part of the anaesthetized mice for 30 s. Thereafter, the acetone/ether cotton was removed and immediately replaced with cotton soaked in distilled water and laid for another 30 s. This procedure was repeated for seven consecutive days and allowed to create dry skin with scales and deep wrinkles. After one week, hydrogel precursors were subcutaneous injected in the wrinkled areas. Based on the good injectability, biocompatibility, and enhanced cell proliferation effect of 200 μL of 10 wt% HAP-0.05 was used for this study. At different time points, the anti-wrinkling progress was monitored by photographing the hydrogel implanted site. Additionally, wrinkle scoring was conducted based on established criteria: 0 denoting the absence of wrinkles, 1 for perceptible wrinkles, 2 for mild and shallow wrinkles, 3 for moderately deep wrinkles, 4 for deep wrinkles with well-defined edges, and 5 for severe and very deep wrinkles accompanied by redundant folds, following previous reports [58]. The mice were sacrificed one week after the treatment, and the skin biopsies were collected to measure the amount collagen deposition and hydroxyproline content.

For histological analysis, skin biopsies were fixed in 10 % neutrally buffered formalin and allowed to incubate for 24 h. Following fixation, the tissues were stained with Masson's trichrome and hematoxylin and eosin (H&E). Finally, the stained sections were analyzed using a confocal laser scanning microscope (A1R; Nikon, Japan).

### 2.16 Quantification of hydroxyproline content

Total amount of collagen in the rejuvenated skins was determined using hydroxyproline assay [59,60]. Briefly, the collected skin biopsies were added to the polycarbonate tube containing 6N HCl and heated to 110 °C for 16 h. Thereafter, the samples were neutralized using NaOH and then kept at 80 °C to dry the samples. The dried powder was suspended in toluene and agitated for 30 min. The suspension was centrifuged and the organic layer containing proline was collected and added to the Ehrlich's reagent in DMSO and the mixtures were incubated for 2 h at 37 °C. Finally, the hydroxyproline content was quantified by measuring the absorbance at 550 nm using microplate reader.

### 2.17 Statistical analysis

The values presented in the data are mean ± Standard Deviation (SD). The statistical significance was calculated using one-way ANOVA test with Tukey's post hoc test. A p value of <0.05 was considered statistically significant value.

## 3 Results and discussion

### 3.1 Synthesis of cross-linked HA

**Fig. 1:**
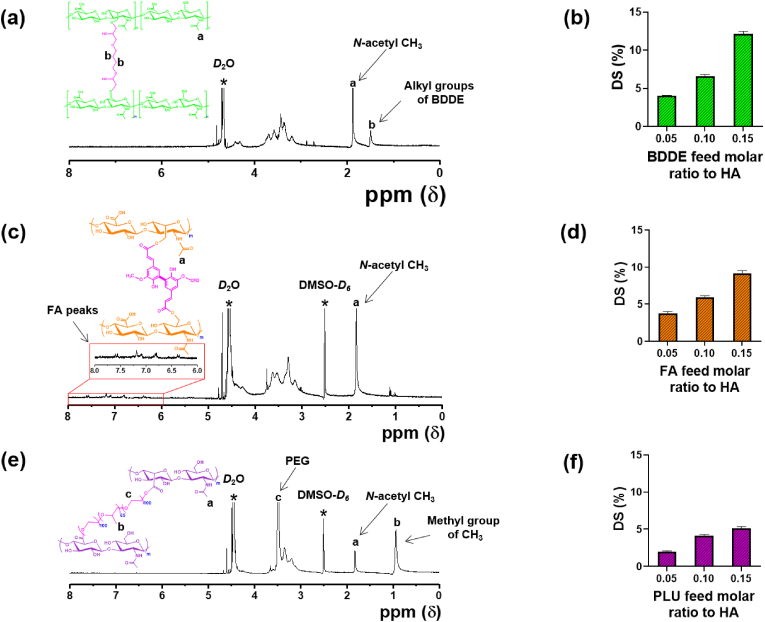
^1^H NMR spectra of HA cross-linked (a) HAB, (c) HAF, and (e) HAP polymers. Degree of modification of (b) HAB, (d) HAF, and (f) HAP polymers.

Scheme 1a shows the synthesis route to prepare HAB for which cross-linker BDDE was reacted with alkaline HA [61]. The chemical structure of representative HAB was characterized using ^1^H NMR spectra. As shown in Fig. 1a, the characteristic peak of *N*-acetyl glucosamine appeared at 1.945 ppm and other peaks corresponding to the anhdyroglucose peaks observed between 3 and 4 ppm. On the other hand, the new peaks of BDDE appeared at 1.598 ppm suggesting the formation cross-linked HAB. The degree of BDDE cross-linking was calculated by comparing the integral ratio of *N*-acetyl peaks of HA with methylene protons of BDDE at 1.598 ppm. The degree of cross-linking increased as the feed ratio of BDDE to HA increased. From the ^1^H NMR spectra, when the feed ratio was increased from 5 % to 15 % the degree of cross-linking of BDDE was increased from 4.1 % to 12.2 % (Fig. 1b). As a result, the molecular weight of the cross-linked HA materials has been increased (Table S1).

The cross-linked HAFs were synthesized using one-pot two-step process as shown in Scheme 1b. Due to the solubility difference between hydrophilic HA and hydrophobic FA, instead of carbodiimide-mediated coupling reaction we employed CDI mediated coupling reaction. Firstly, the FA was activated using CDI in DMSO and were grafted onto the backbone of HA through esterification reaction. Upon increasing the feed ratio of FA to HA, yellowish HAFs are obtained and the color getting stronger as the feed ratio increased. Successful conjugation was verified using ^1^H NMR spectra (Fig. 1c). ^1^H NMR spectra depicts the resonance signals of FA at 6.60–7.60 ppm along with the presence of *N*-acetyl glucosamine at 1.945 ppm. The degree of substitution of FA to HA was calculated by comparing the aromatic integral signal of FA with *N*-acetyl glucosamine of HA. As expected, the degree of substitution increased as the feed ratio of FA to HA increased (Fig. 1d).

As shown in Scheme 1c, cross-linked HAP was synthesized by activating the HA using CDI followed by mixing with solution of PLU in DMSO under mild heating conditions. From this study, CDI has been found to be an efficient activator of polysaccharides, including HA. By varying the feed molar ratios of PLU cross-linker to HA disaccharide repeating units, three different cross-linked HAP was prepared. The chemical structure and cross-linking degree were confirmed using ^1^H NMR spectra (Fig. 1e). Appearance of new characteristics peaks at 0.98 and 3.56 ppm confirmed the presence of PLU and its integration value was compared with native HA peak of *N*-acetyl glucosamine at 1.86 ppm. As shown in Fig. 1f, the degree of cross-linking of PLU was attained a plateau after the feed molar ratio reached 15 %. This can be explained due to the high molecular weight nature of PLU that restricts movement of molecules due to the molecular entanglement.

### 3.2 FT-IR analysis of cross-linked HA

**Fig. 2:**
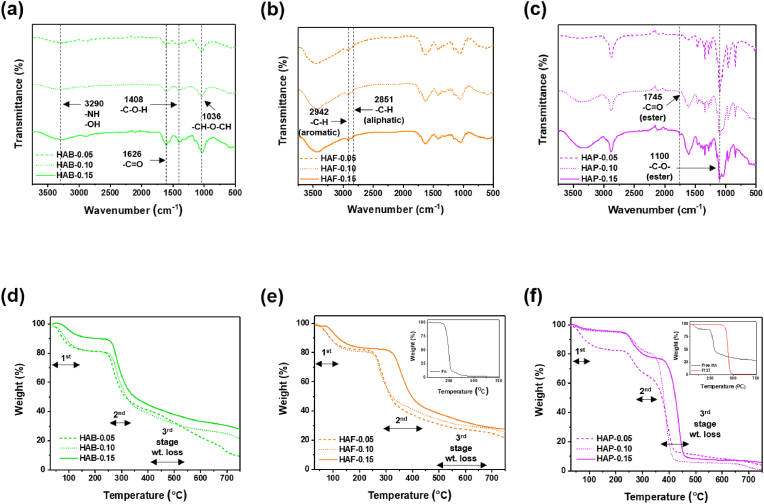
FT-IR and TGA curve of (a, d) HAB, (b, e) HAF, and (c, f) HAP polymers.

FT-IR spectroscopy was used to characterize the chemical structure and compositions of cross-linked HA. Regardless of cross-linking agents, the FT-IR spectra shows the broad characteristic band at 3290 cm^−1^, which is attributable to the hydroxyl, carboxyl and amide bonds in the HA. In addition, the carbonyl stretches –C=O of amide groups appeared at 1626 cm^−1^, and the C-OH bending vibrations appeared at 1408 cm^−1^. In addition to these characteristic bands, the bands corresponding to the cross-linkers were also observed. For HAB formulation, the ether stretches C–O–C vibration band at 1036 cm^−1^ gradually increased as the feed ratio of BDDE to HA increased because the hydroxyl groups in the HA transformed into ether linkages during the cross-linking process (Fig. 2a).

FT-IR spectra of HAF showed a mild shifting of the C=O stretching band at 1730 cm^−1^, implying the ester formation between carboxylic acid groups FA and hydroxyl groups of HA (Fig. 2b). 10.13039/100014337Furthermore, the peaks at 940 cm^−1^ indicated the presence of alkene (C=C-H) that supports the FA conjugation to the backbone of 10.13039/100031353HA. Similarly, the FT-IR spectra of HAP was recorded to investigate the successful conjugation and is shown in Fig. 2c. The intense band of C–O–C corresponding the ether groups of PEG present in the PLU appeared at 1100 cm^−1^. In addition, the intensity of ester band increased as the feed ratio of PLU to increased, which indicated the successful cross-linking via ester bond formation.

### 3.3 Thermal properties

Thermal stability and inter- and intramolecular interactions of the materials was investigated using TGA analysis [62]. Thermograms of all the cross-linked materials with different degree of substitution is shown in Fig. 2d–f. From the thermograms, it is clear that cross-linked HA materials showed increased thermal stability and the extent of stability increased as the cross-linking degree increased. This is mainly due to formation of a highly cross-linked network and strong inter- and intramolecular interactions with the network.

Precisely, the weight loss of the materials in the temperature range between 50 °C and 750 °C can be divided into three stages. The first stage weight loss is due the loss of water molecules bound in the hydrophilic segments of the biomaterials. Upon increasing the temperature from 50 °C to 140 °C, the weight loss in the materials was varied from 5 to 14 % depending on the cross-linkers and cross-linking density. Among three different materials, extent of thermal stability was significantly higher in HAB materials. Upon gradually increasing the temperature, the materials enter into the second stage weight loss. In this stage, the weight loss occurred by the decomposition of functional groups in the cross-linkers as well as HA. Functional groups in the HA such as -COOH, -OH, and -NH_2_ are easily decomposed, which resulted in high weight reduction [63]. In particular, the extent of reduction was high in HAP conjugates with the weight loss of over 80 % at 450 °C. On the other hand, for HA cross-linked with small molecule cross-linkers, like BDDE and FA, the degradation rate was lower than that of HAP materials. This may be due to the strong intermolecular interactions of the network can restrict polymer chain movement, which reduce the decomposition rate [64]. From thermal analysis results, it is evident that after chemical cross-linking thermal properties of HA was significantly improved.

### 3.4 Sol-to-gel phase transition and extrusion properties

Sol-to-gel phase transition property of the hydrogels was measured via a vial tilting method by visualizing the flowability of the gels [65,66]. The gel property of the materials was examined by employing temperature and enzyme stimuli. PLU is a triblock copolymer exist as free-flowing sols at low temperature and form hydrogel network upon raising the temperature [67]. At low temperature, PLU solutions exists as unimers with low-viscosity that transformed into a self-assembled micelle aggregates of immovable hydrogels at high temperature [68]. FA is an amber colored phenolic phytochemical susceptible to oxidize in the presence of laccase [69], which enables the formation of cross-linked networks. Therefore, sol-to-gel phase transition of HAB, HAF and HAP was examined by introducing appropriate stimuli.

**Fig. 3:**
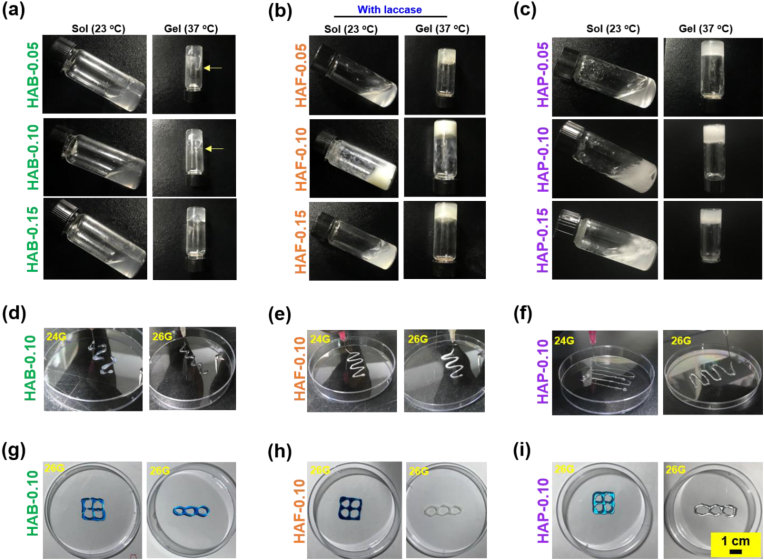
Sol-to-gel phase transition property of cross-linked (a) HAB, (b) HAF, and (c) HAP polymers. The phase transition was investigated by varying the temperature from 0 to 37 °C. In case of HAF polymer, laccase was added and incubated at 37 °C. Extrudability and printability of (d, g) HAB, (e, h) HAF, and (f, i) HAP polymers.

As expected, HAB materials doesn't show any thermo-responsiveness because of the absence of appropriate temperature-sensitive functional polymers (Fig. 3a). However, flowability of the HAB materials was reduced as the cross-linking density of the HAB materials increased. In particular, HAB-0.15 showed weak gel formation due to high cross-linking density. In case of HAB-0.05, flowing rate was higher when compare with other two formulations. Interestingly, HAF materials form stable gel network upon exposure with laccase (Fig. 3b). Laccase is a multicopper oxidase that catalyze the formation of phenoxy radical and induce intermolecular cross-linking [70]. For cross-linking of HAFs, laccase enzyme (10 U/mL) was mixed with 5 wt% HAF conjugates. Regardless of the degree of substitution of FA in the backbone HA, all three HAF materials form stable hydrogel network. It should be noted that, at higher conjugation ratio (i.e., HAF-0.15) due to the high concentration of lipophilic FA restricts the moment of HAF materials and shown poor flowability even before cross-linking.

As shown in Fig. 3c, the HAP materials exhibited sol-to-gel transition between low temperature (0 °C) and body temperature (37 °C). Increase of PLU content in the HAP materials reduces the gelation time. As demonstrated in Fig. S1, the gelation times gradually decrease with increasing PLU content in the HAP materials. For instance, the HAP-0.15 showed rapid gelation when compared with HAP-0.10 and HAP-0.05 (Fig. S1). This is due to the greater number of triblock PLU in the materials induce the micelle packaging at the physiological condition [71]. From the sol-to-gel test, it's clear that HAF and HAP exhibit phase transition in response to enzyme and temperature, respectively. Although HAB materials does not show change in phase transition properties, the HAB materials showed some viscous properties and this kind of materials can be utilized for the topical treatment of skin-related diseases. The temperature-dependent sol-gel phase transition of HAP was confirmed by examining its rheological properties, specifically through measurements of viscosity and modulus changes with temperature.

The rheological properties of hydrogels play a pivotal role in both in vivo and in vitro injectability. To investigate the rheological characteristics of HAP-0.05 and PLU hydrogels, we examined their complex viscosity and modulus (G′ and G″) at various temperatures. As anticipated, the viscosity of both HAP-0.05 and PLU hydrogels increased with rising temperature (Fig. S2a). Furthermore, we determined the storage modulus (G′) and loss modulus (G″) to validate gelation. Fig. S2b illustrates the typical elastic behavior observed when the temperature reached room temperature, gradually increasing until reaching a plateau under physiological conditions. This behavior suggests the prevalence of the materials' elastic nature over their viscous nature.

Furthermore, to investigate the extrudability, hydrogel precursors were transferred into syringe barrel equipped with different needle sizes. Although both HAF and HAP materials exhibit good gelling properties, we chose HAF-0.10 and HAP-0.10 to examine the extrusion properties as these materials are shown to possess good hydrophobic-hydrophilic balance and good gel formation. In addition, we briefly examine the extrudability of HAB-0.10. As shown in Fig. 3d–f, HAF and HAP materials were continuously extruded through both 24G and 26G needles. However, HAB materials difficult to extrude in small needles (24G) and required high injection force for gels to flow, which also showed discontinues extrusion with aggregation. Interestingly, the extrusion was better in 26G needles. It should be note that, HAB, HAF, and HAP could smoothly extrude and write defined structures in 26G needles (Fig. 3g–i). Moreover, the injectability rating of HAB, HAF, and HAP hydrogels was qualitatively assessed according to previous reports [72], and the details are presented in Table S2.

### 3.5 Microscopic structure of freeze-dried hydrogels

**Fig. 4:**
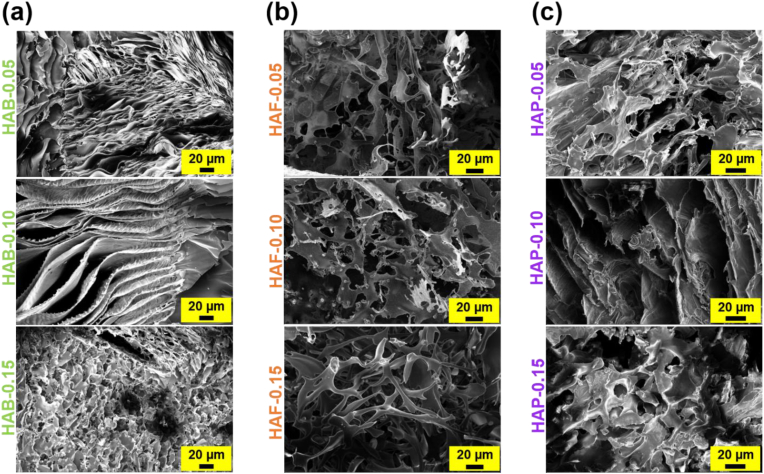
SEM micrographs of (a) HAB, (b) HAF, and (c) HAP hydrogels with different degree of cross-linking density.

Porous morphological properties of hydrogels are very important as it can impact the applications of hydrogels in various aspects [73]. It is known that porous properties of the hydrogels influence the mechanical properties of hydrogels. The morphology of HA-based hydrogels prepared with different cross-linking agents and density is shown in Fig. 4. Regardless of cross-linkers and their density, all the hydrogels shown porous properties. By increasing the cross-linking density, the pore size of the hydrogels decreased. At higher cross-linking ratio (0.15), the pore size of the hydrogels was decreased when compared with hydrogels prepared at 0.05 and 0.10 M cross-linking ratio. In particular, the hydrogels prepared at high cross-linking density are well inter connected and most of the hydrogels prepared at this ratio exhibited honeycomb-like structures. It should be noted that cross-linking agents BDDE had little effect in topological structure of hydrogels when compared with FA and PLU cross-linkers. SEM analysis suggests that the HAB exhibits a lamellar morphology, which results from the interplay between the hydrogel's intrinsic structure and the lyophilization process. Morphological analysis of freeze-dried hydrogels demonstrated that porous nature of the hydrogels could effectively swell in physiological fluids and retain significant fraction of fluids within the hydrogel network [74]. The porous properties of freeze-dried hydrogels could be beneficial in cell proliferation as it can facilitate the exchange of water, gas, nutrient and metabolite during the cell growth [75].

### 3.6 Mechanical properties of hydrogels

**Fig. 5:**
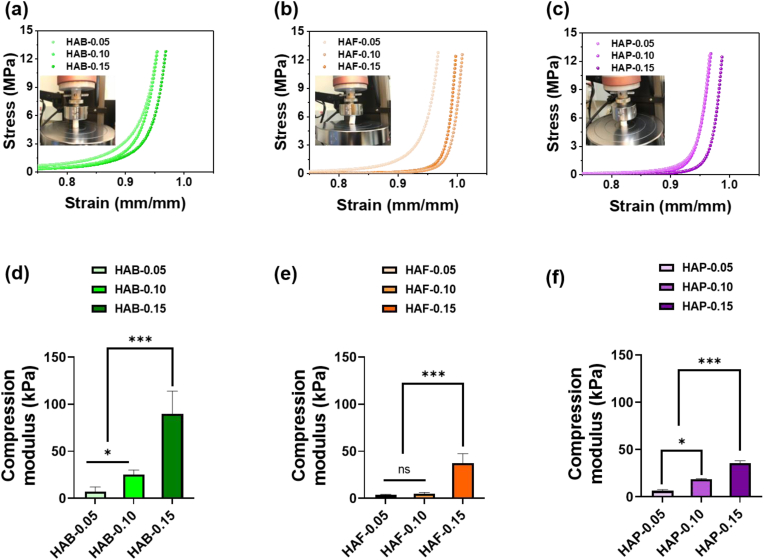
Compressive stress-strain curve of (a) HAB, (b) HAF, and (c) HAP hydrogels with different degree of cross-linking density. The compressive modulus of (d) HAB, (e) HAF, and (f) HAP hydrogels. Asterisks (∗) denotes statistically significant differences calculated using one-way ANOVA test with Tukey's post hoc test. ∗∗∗P < 0.001 and “ns” indicates not significant.

To examine the effect of cross-linkers on mechanical properties, cylindrically shaped HAB, HAF and HAP hydrogels composed of different density of cross-linkers were prepared. In general, physically cross-linked polysaccharide hydrogels with brittle network structure often exhibited weak mechanical strength because the networks formed by hydrogen bonding and weak van der waals forces [76]. As shown in Fig. 5, upon increasing the cross-linking density in the HA network, the toughness of the hydrogels increased. In particular, HAF hydrogels exhibited higher toughness than HAB that can be explained by the FA cross-linking in the network. It has been reported that cross-linking proteins by FA increased the emulsion and rheological properties [77]. Interestingly, HAP hydrogels exhibited superior toughness when prepared with high cross-linking density. Among three hydrogel groups, HAP exhibited superior mechanical properties. The PLU polymers in the hydrogel network form more hydrogen bonds which strengthen the hydrogel matrix. Specifically, there was an increase in mechanical properties as the degree of cross-linking rose. Moreover, upon comparing the mechanical properties of different formulations, it was observed that the HAB formulation exhibited superior mechanical properties, while HAF and HAP hydrogels demonstrated a decrease. The order of mechanical properties, accordingly, was HAB > HAP > HAF. In this study, the compressive modulus was calculated in the range of 10–20 % strain to better reflect the deformation behavior of the hydrogels under conditions relevant to their intended applications in soft tissue engineering [53]. This range was chosen because the hydrogels are designed to withstand moderate compression, which is more representative of physiological conditions such as those experienced in dermal or subcutaneous tissues. However, it is also important to consider how hydration affects the mechanical performance of these hydrogels, as their structural integrity and functionality can vary depending on their water content.

In general, the compression modulus of dried hydrogels is higher because the polymer network is dense and rigid in the absence of water molecules. Additionally, without water, strong intermolecular interactions, such as hydrogen bonding and van der Waals forces between polymer chains, make the material stiffer. In contrast, hydrated hydrogels exhibit a lower compression modulus because the water absorbed into the polymeric network acts as a plasticizer. The swelling of the hydrogel increases the distance between polymer chains, reducing intermolecular interactions and making the structure more compliant. Consequently, the presence of water enhances polymer chain mobility, allowing the hydrogel to deform more easily under compressive stress. As shown in Fig. S3, the compression modulus of dried HA hydrogels was higher than that of their hydrated counterparts due to the absence of water, which influences the network's rigidity. Regardless of hydration state, the HA-based hydrogels prepared in this study exhibited a compression modulus in the range of 10–100 kPa, demonstrating a balance between stiffness and flexibility. This mechanical profile suggests their suitability as dermal fillers for soft tissue engineering and wound healing applications.

### 3.7 Hemocompatibility of hydrogels

**Fig. 6:**
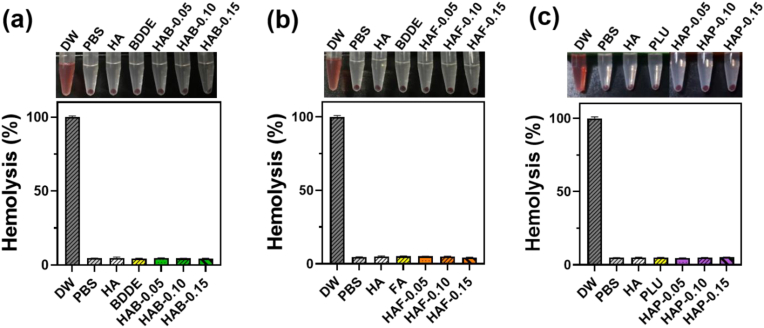
Hemolysis ratio of (a) HAB, (b) HAF, and (c) HAP hydrogels with different degree of cross-linking density. PBS was used as the negative control and DW was used as the positive control. The data in the graph presented as mean ± SD (n = 3).

Chemical modification of HA may cause morphological changes of RBCs or rupture of erythrocyte membranes, which lead to the hemolysis [78]. Since blood contact inevitable for hydrogels used in biomedical applications, it's important to evaluate the blood compatibility of hydrogels in contact with RBCs [79]. Hemolysis of the hydrogels was calculated by measuring the absorbance of hemoglobin released after the lysis of RBCs [80]. Hydrogels with different cross-linkers and cross-linking density were set as experimental groups for this study, and DI water and PBS were served as positive and negative control, respectively (Fig. 6). As expected, DI water control group transformed into bright red color due to the rupture of RBCs. Interestingly, the supernatant of all the hydrogel was colorless with pellets of RBCs at the bottom of tube, similar to the PBS negative control group. Quantitative analysis also followed the similar trend, indicating the good hemocompatibility of the hydrogels. It should be noted that, hemolysis of HAP hydrogel groups was less than 5 %, which suggests the suitability of HAP hydrogels in clinical applications. Although HAB and HAF hydrogels found to be safe, supernatants of HAB hydrogels showed pale pink color, which requires further investigation before applying to clinical applications.

### 3.8 Biocompatibility of hydrogels

**Fig. 7:**
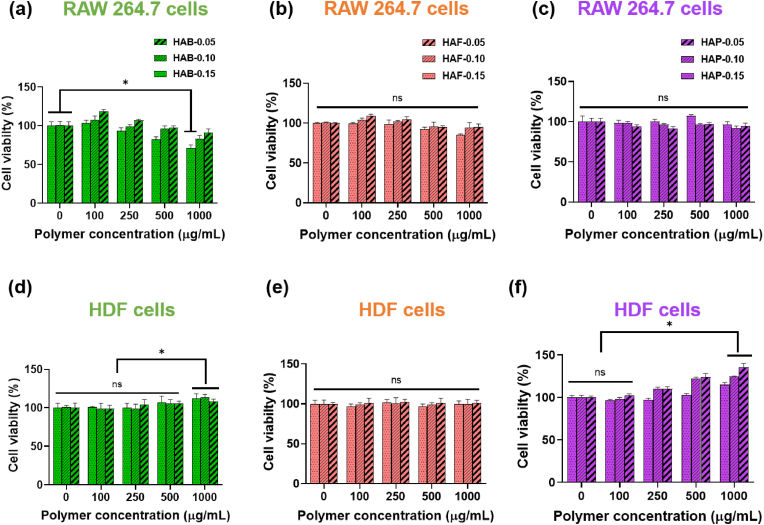
Cell viability of RAW 264.7 and HDF cells treated with different concentration of HA hydrogels possessing different cross-linking density for 48 h. Asterisks (∗) denotes statistically significant differences calculated using one-way ANOVA test with Tukey's post hoc test. ∗P > 0.05 and “ns” indicates not significant.

In addition to the hemocompatibility, hydrogels used in the biomedical applications should have good biocompatibility, which supports the host cell interactions that are beneficial in tissue engineering applications [81]. To examine the biocompatibility of hydrogels, in vitro cytotoxicity of HA hydrogels was assessed using MTT assay by measuring the cellular metabolic activity of RAW 264.7 and HDF cells after exposing with hydrogel precursors. As shown in Fig. 7a-c, the different HA-based hydrogels prepared in this study showed no toxic effect and the cell viability of these hydrogel treated groups were similar to the untreated control groups cultured with only media. The RAW 264.7 cells treated with HA hydrogels exhibited maximum metabolic activity, which implied the safety of the HA based hydrogels.

Furthermore, the biocompatibility of hydrogels was tested using HDF cells as these types of cells directly involved in the soft tissue modification process. Viability of HA hydrogels incubated with HDF cells is shown in Fig. 7d–f. The results revealed that HA based hydrogels found to be non-toxic to the HDF cells. In particular, the cell viability of HAB and HAP hydrogels treated groups was higher than that of untreated control group. This indicated the growth and proliferation effects of HAB and HAP on HDF cells. Notably, HAP significantly enhanced fibroblast growth by 35.6 %, which implied that HAP hydrogels can be a suitable material for fast wound closure.

**Fig. 8:**
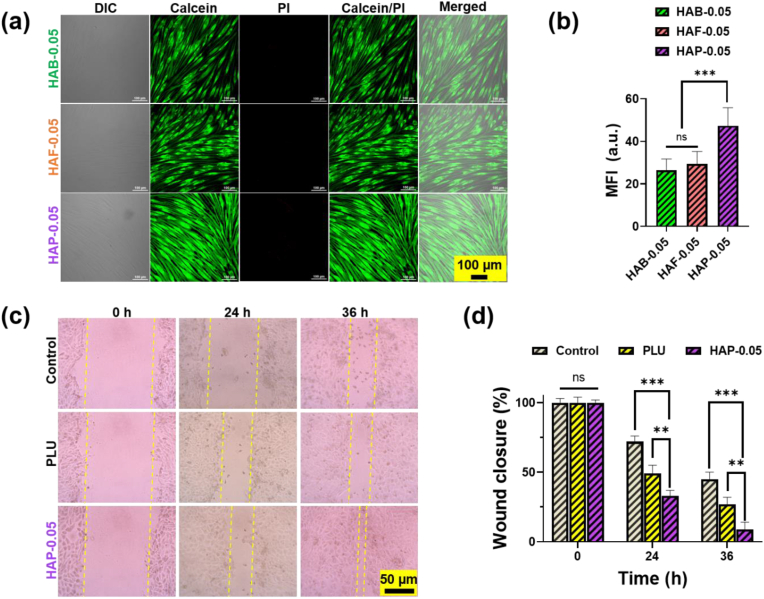
(a) Live/Dead confocal fluorescent microscopic images of HDF cells treated with HA cross-linked hydrogels. Untreated control group received only cell culture medium treatment was served as control. Green fluorescence (Calcein) depicts live cells, while red fluorescence (PI) depict dead cells. (b) MFI of green fluorescence cells; the values are for Mean ± SD three individual panels. (c) In vitro scratch wound healing assay of HaCaT cells co-cultured with different hydrogel formulations. The HaCaT cells received only culture medium was served as control. (d) Wound contraction rate of HaCaT cells treated with hydrogels. Asterisks (∗) denotes statistically significant differences calculated using one-way ANOVA test with Tukey's post hoc test. ∗P > 0.05; ∗∗∗P < 0.001; “ns” indicates not significant.

The enhanced biocompatibility of hydrogels was further tested using Live/Dead viability assay. After 24 h, the HDF cells treated with HAP hydrogels were stained using a Live/Dead mammalian cell viability kit. The fluorescent images of HDF cells are shown in Fig. 8a and b. Cell micrographs of HDF cells showed dense monolayers for HAP treated cells. The cells treated with other hydrogels also maintained the good cell integrity. The Live/Dead cell imaging results agree with the cell viability test, indicating the safety of the hydrogels to cells. Taken together, the cell test suggested the possibility to integrate HAP hydrogels into an injectable biocompatible hydrogel capable of inducing cell proliferation in fibroblast cells.

### 3.9 HaCaT cell growth and scratch healing in the presence of hydrogels

To further investigate the enhanced cell proliferation, cell migration study of HaCaT cells was performed by co-culturing with hydrogel formulations in a time-dependent manner. Using the scratch healing assay, researchers can assess how effectively hydrogel formulations accelerate the closure of simulated wounds in vitro. If a hydrogel formulation demonstrates faster wound closure in the assay, it indicates potential efficacy in promoting skin repair and regeneration. This promising result suggests that the hydrogel may also have anti-wrinkling effects in vivo, as improved skin repair and regeneration contribute to overall skin health and resilience against aging-related changes.

To examine the cell migration, we employed an in vitro scratch healing technique in which rate of wound closure was calculated on scratch induced confluent monolayer cells. In order to examine the scratch healing potential, a linear scratch was created on confluent cell monolayer of HaCaT cells. After incubating with hydrogels, the HAP hydrogels treated group showed enhanced motility and migration in comparison with PLU hydrogel and control groups (Fig. 8c and d). The cell migration study result demonstrated that HAP hydrogels found to be safe and biocompatible and could effectively enhance the migration of cells.

### 3.10 Effect of hydrogels on MAPKs and MMPs expression

In skin aging and wrinkling, increased secretion of MMPs by ROS are the major factors [82]. In skin, ROS are developed from both extrinsic and intrinsic sources by ultraviolet irradiation and metabolic pro-oxidants (Scheme 1d) [83]. Upon ROS generation, the family of MAPK is activated including ERK, p38, and JNK (Scheme 1e) [84]. This led to the activation of transcription factor, activator protein 1 (AP-1), an essential protein in the transcriptional regulation of MMPs. MMPs are a family of zinc-dependent endopeptidases that degrade components of the ECM [85,86]. In general, the UVB irradiation and age factors induce the synthesis of MMPs in skin cells, including fibroblasts and keratinocytes, which in turn causes the abnormal turnover of ECM component and promotes skin wrinkling [87]. Particularly, MMPs are responsible for the degradation of various ECM proteins including collagen, elastin, fibronectin and proteoglycan. Therefore, increased expression of MMPs are responsible to the connective tissue damage in skin aging and photodamage. Therefore, inhibition of MAPKs and downregulating the expression of MMPs is an effective strategy to overcome skin damage and skin wrinkling induced by UVB irradiation.

**Fig. 9:**
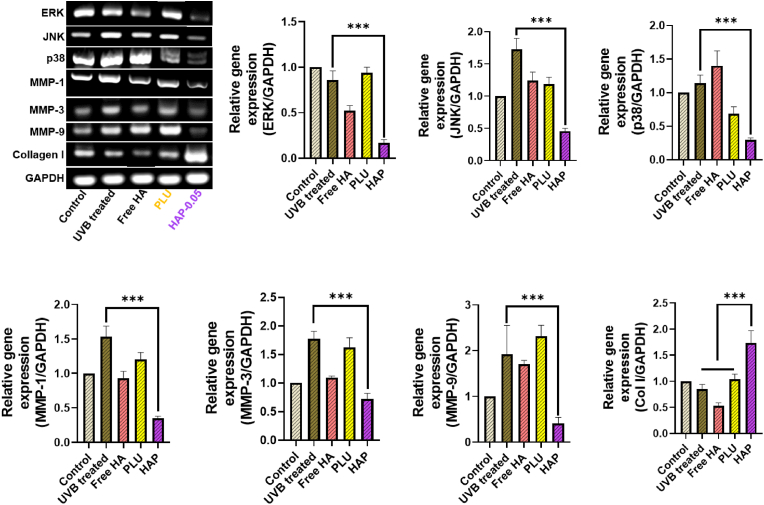
RT-PCR analysis of mRNA extracted from HDF cells treated with different hydrogel formulations (HA, PLU, HAP) and UVB irradiation. The relative gene expression levels of MAPKs (ERK, JNK, p38), collagen-related genes, and MMPs (1, 3, 9) were quantified using ImageJ. Asterisks (∗) indicate statistically significant differences (p < 0.001) determined by one-way ANOVA followed by Tukey's post hoc test.

To examine the correlation between MAPKs and collagen expression, we first assessed the effect of hydrogels on MAPK expression in UVB-irradiated HDF cells, using RT-PCR analysis. As expected, UVB irradiation induced phosphorylation of various MAPKs, including ERK, JNK, and p38 (Fig. 9), which subsequently led to a significant upregulation of MMP expression. However, HDF cells treated with HA and PLU hydrogels exhibited a mild reduction in the phosphorylation of MAPKs. Interestingly, the HAP hydrogel group effectively suppressed the phosphorylation of ERK, JNK, and p38 (Scheme 1f), which could subsequently reduce MMP secretion. Specifically, we propose that the HA and PLU components in the HAP hydrogel, due to their macromolecular structure and potential to form a hydrated, biocompatible environment, create favorable conditions for cellular signaling. This environment may reduce cellular stress and inhibit the activation of MAPK pathways (ERK, JNK, p38), which are commonly associated with collagen degradation. Furthermore, the suppression of MMPs (MMP-1, MMP-3, and MMP-9) could be attributed to the anti-inflammatory effects of the hydrogels and the stabilization of ECM components provided by the HAP hydrogel. As a result, there was a significant enhancement in collagen expression in the HAP hydrogel group. Additionally, quantitative analysis of the investigated genes, normalized to control cells, confirmed the upregulation of collagen expression in the HAP hydrogel group.

Furthermore, we have conducted RT-PCR analysis for pro-inflammatory cytokines (IL-6 and TNF-α) using HaCaT cells to assess the inflammatory response of the hydrogels. The results indicate that the HAP-0.05 hydrogel exhibits minimal inflammatory activation, supporting its biocompatibility (Fig. S4).

### 3.11 Hydrogels suppressed MMPs expression in UVB-irradiated cells

To explore the molecular mechanisms underlying the suppression of the MAPK signaling pathway, we investigated the protein expression of MMPs using Western blot analysis (Fig. S5). Our results revealed that HAP hydrogels effectively downregulated MMP-3 expression in a concentration-dependent manner. In contrast, both PLU and free HA treatments did not result in any suppression of MMP-3 expression; their Western blot bands were comparable to those of the untreated control groups. It is well-established that UVB irradiation induces the production of cytokines, which activate fibroblasts and subsequently increase MMP production, leading to collagen degradation. Our findings suggest that HAP hydrogel treatment inhibits collagen degradation by suppressing MMP expression, thus providing a potential mechanism for the hydrogel's protective effects on collagen integrity.

### 3.12 In situ gelation of hydrogels in vivo

**Fig. 10:**
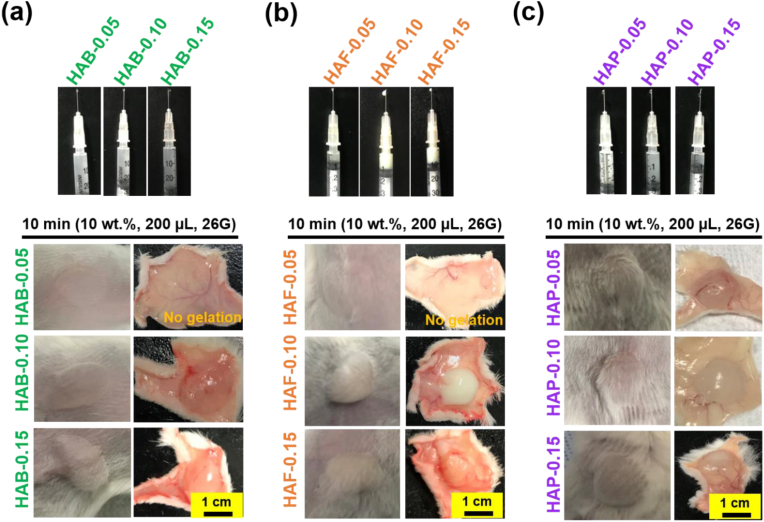
(a, b and c) Injectability test of HA based cross-linked hydrogel precursors. The precursors are injected subcutaneously into the back of BALB/c mice to examine in vivo gel formation. Images are captured during implantation and gels are recovered 30 min after the formation of gels.

Subcutaneous injection technique was employed to investigate the in situ in vivo gel formation of hydrogels [88]. Prior to injection, hydrogel precursors were transferred to hypodermic syringes equipped with 26G needles and extruded to examine the flowability of the hydrogels. As shown in Fig. 10, all the hydrogel precursors extruded easily from the 26G needles, which indicated that hydrogel precursors are low viscous at low and room temperature. Upon subcutaneous injection, materials cross-linked with higher cross-linking density (0.10 and 0.15) form stable viscoelastic hydrogels at the dermis layers of BALB/c mice. It should be noted that HAB and HAF prepared at lower cross-linking density (0.05) not formed gels. During the hydrogel implantation, no evidence of infection was observed, which implied the good safety of the hydrogels. In particular, HAP hydrogels showed sharp sol-to-gel phase transition and form gels right after injection. This is mainly due to the thermo-responsive property of PLU in the HAP as observed in the previous test. Although HAF materials formed gels, it took 10–20 min to obtain stable gel. It should be noted that phase transition property of the HAB materials not altered after injection but it showed some stability at the dorsal region. To evaluate biodegradability, HAP-0.05 was chosen as the representative hydrogel due to its favorable attributes such as smooth injectability, biocompatibility, and enhanced cell proliferation. PLU served as the control in this assessment.

Before in vivo applications, we examined the swelling properties of the hydrogels, as high swelling rates in dermal filling applications may exert significant pressure on surrounding tissues. The results presented in Fig. S6 and Table S3 demonstrate a correlation between the swelling ratio and crosslinking density. The swelling trend indicates that as the crosslinking agent feed concentration increases, the equilibrium swelling ratio decreases, while the effective crosslink density of the polymer network correspondingly rises. BDDE, a small molecule cross-linker, contributes to high stiffness and stability due to its compact structure and dense network formation. FA, containing a phenolic structure, imparts antioxidant properties and allows for enzyme-mediated sol-to-gel transitions, making it suitable for controlled delivery. PLU, a macromolecular cross-linker, provides tunable gelation behavior via temperature sensitivity, enhances injectability, and promotes cell viability. These structural variations directly influence the physicochemical properties of the hydrogels, such as injectability, enzymatic and thermal responsiveness, and biocompatibility.

Notably, BDDE shows a significantly lower crosslinking density compared to FA, whereas Pluronic F127 exhibits a markedly higher crosslinking density. This difference can be explained by the properties of F127, a high-molecular-weight polymer whose crosslinking mechanism is driven by temperature changes. When the temperature exceeds its LCST, the polypropylene oxide (PPO) segments in the PLU support micelle formation, resulting in a dense crosslinking network and minimal changes in swelling, given the reduced hydrophilicity at 37 °C. In contrast, the other two crosslinkers have considerably lower molecular weights, with BDDE showing relatively slow reaction kinetics, often making it challenging to form gels at low crosslinking densities. Thus, the cross-linking density (ν_e_) values for low cross-linking density HAB samples align with the hypothesis. The incorporation of natural polymers into PLU effectively regulated swelling, consistent with findings from prior studies [89,90].

To illustrate controlled biodegradation property of hydrogels, we implanted the hydrogel into the backs of mice and sacrificed the mice at various time intervals to assess the condition of the hydrogels. For this study, we subcutaneously injected 200 μL of HAP (10 wt%), PLU (10 wt%), and PLU (20 wt%) hydrogel precursors into the dorsal region of BALB/c mice. When only PLU hydrogel (20 wt%) was injected in situ, rapid biodegradation was observed, with the PLU gels completely disappearing within 24 h (Figs. S7a and S7b). Conversely, 10 wt% PLU hydrogels failed to form an in-situ gel. Remarkably, the 10 wt% of HAP-0.05 formulation formed a stable hydrogel depot exhibiting controlled biodegradation. Approximately 60 % of the hydrogel mass reduced after 7 days, and after three weeks, ∼10 % of the hydrogel remained intact (Fig. S7c). This observation underscores the controlled biodegradability of HAP hydrogels, rendering them suitable for applications such as soft tissue engineering. The controlled biodegradability of HAP hydrogels confers the ability to sustain collagen production.

### 3.13 Anti-wrinkling property of hydrogels in vivo

**Fig. 11:**
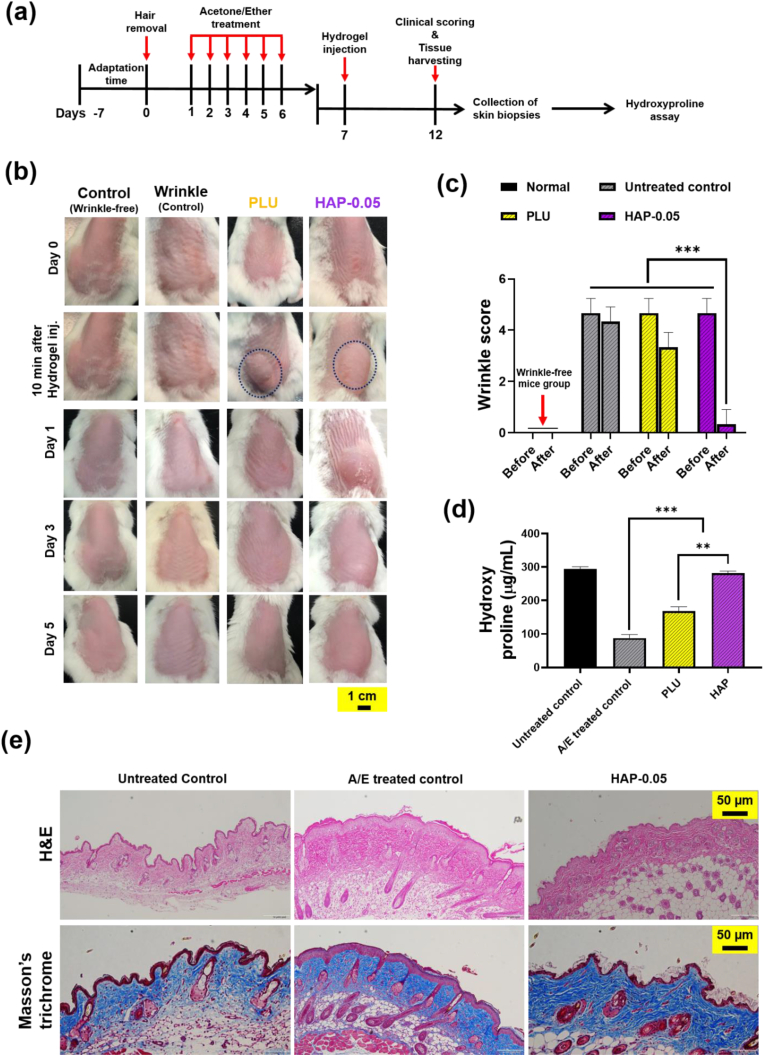
(a) Experimental set up for the preparation of skin wrinkling mice model and their treatment schedule. (b) Skin surface images of the mice after hydrogel treatment and the mice received without treatment was served as control (n = 3). (c) Wrinkle scoring evaluation before and after hydrogel injection (n = 3). (d) Amount of collagen deposition from skin tissues (n = 3). (e) H&E and Masson's trichrome staining images of untreated control, A/E treated control and HAP-0.05 hydrogel groups. Asterisks (∗) denotes statistically significant differences calculated using one-way ANOVA test with Tukey's post hoc test. ∗∗P > 0.01; ∗∗∗P < 0.001; “ns” indicates not significant.

The HAP hydrogels prepared in this study showed good hemocompatibility, enhanced proliferation in HDF cells, and easy gel formation in vivo can be suitable materials for soft tissue augmentation. The anti-wrinkling property of the HAP hydrogels was tested in BALB/c wrinkle model prepared by the treatment of acetone/ether mixture (1:1) by following previous reports [91]. Mice treated with acetone/ether mixture showed dry skin and formation of wrinkles, which confirmed the formation of wrinkles in the mice (Fig. 11a–c). To examine anti-wrinkling effect, HAP hydrogels were subcutaneously injected into the wrinkle region and their efficacy was evaluated. The control sample group, which received no treatment, showed skin roughness and wrinkling. Interestingly, HAP injected hydrogel group significantly reduced skin roughness and reduced the wrinkles, indicating the anti-wrinkling property of HAP hydrogels. In particular, from the skin surface images of the mice, it was observed that 3 days after injection, PLU hydrogels exhibited more wrinkles compared to HAP gels. However, interestingly, after 5 days, the wrinkles in the HAP gel group were completely recovered, while the PLU gel group still showed wrinkles (Fig. S8). From these results, it is concluded that HAP materials could be a suitable material for anti-wrinkling applications. Next skin biopsies were collected and collagen content in the skin samples were analyzed. Materials employed in anti-wrinkling applications should increase the collagen production in the skin [92,93].

### 3.14 Effect of HAP hydrogels on collagen deposition

It is known that hydroxyproline is the major component of collagen and therefore determination hydroxyproline content in the skin tissues directly reflect deposition of collagen. As expected, the acetone-ether treated group less amount of hydroxyproline compared with normal mice group (Fig. 11d). This is due to the loss of collagen synthesis caused by the solvent treatment. Interestingly, HAP hydrogel group exhibited significant increment in hydroxyproline content when compared with PLU and control group. The significant difference in the hydroxyproline content suggest that the injectable HAP hydrogels could promote collagen secretion.

Collagen holds a pivotal role in preserving the skin's structure and elasticity [93]. As a fundamental component of the extracellular matrix, it furnishes the skin with strength and support. In the natural aging process, wrinkles emerge, often linked to diminished skin elasticity and firmness attributed to reduced collagen levels. Throughout aging, collagen production in the skin diminishes, and the existing collagen may undergo disorganization. This decline in collagen, coupled with alterations in its structure, contributes to a loss of skin firmness and elasticity, thereby fostering wrinkle formation. Adequate collagen levels are integral for resilient skin, mitigating the likelihood of fine lines and wrinkles. Consequently, our focus with the HAP hydrogel formulation is to sustain healthy collagen levels. The subcutaneous injection of HAP hydrogels effectively stimulates collagen production and reinforces the existing collagen structure. This, in turn, enhances skin elasticity and diminishes the visible signs of wrinkles. This was further verified using histological analysis.

Epidermal hyperplasia is a key parameter in histological in assessing skin damage, as increased epidermal thickness contributes to skin roughness (Fig. 11e). The untreated control group exhibited low epidermal thickness compared to the BALB/c wrinkle model induced by treatment with an acetone/ether mixture (1:1), which resulted in significant epidermal thickening. Interestingly, treatment with HAP hydrogel in the wrinkle models led to a significant reduction in epidermal thickness, comparable to that of the untreated control group. During the aging process, there is a decrease in dermal collagen density, and degradation of collagen fibers is observed in aged skin. Additionally, skin samples stained with Masson's trichrome revealed an increase in collagen bundles in HAP hydrogel-treated mice groups compared to the control group. Therefore, our pursuits geared towards promoting optimal collagen levels to support skin health and combat the effects of aging. Furthermore, histological examination of the hydrogel-implanted site (Fig. S9) revealed minimal immune cell infiltration, further supporting the biocompatibility of the HAP-0.05 hydrogel. In particular, the counts of inflammatory cells in the hydrogel group were similar to those in untreated control mice that did not receive any implantation.

## 4 Conclusions

In summary, a series of HA hydrogels with three different cross-linkers (e.g., BDDE, FA and PLU) with varying degrees of cross-linking density have been synthesized and their physiochemical properties were optimized for cosmetic and anti-wrinkling applications. The synthesis of HA hydrogels was obtained in a one-pot process without any catalyst, resulting in a clean biomaterial that can be suitable for cosmetic applications. The ^1^H NMR spectra were used to quantify the degree of cross-linking, while the characteristics functional groups in the biomaterials were characterized using FT-IR spectra. Upon increasing the cross-linking density in the materials, the HA hydrogels become very tough and showed enhanced mechanical properties. The HA hydrogels showed good hemocompatibility, and biocompatibility to RAW 264.7 and HDF cells. In particular, HA hydrogels stimulated the growth of HDF cells. Co-culturing of HA hydrogels alleviate skin photoaging in UV irradiated HDF cells. RT-PCR results of UVB irradiated HDF cells showed effective suppression of MMPs overexpression and increased secretion of Collagen I, which implied the remodeling of the ECM. The HA hydrogels are smoothly injected into the subcutaneous layers and formed stable gel into the back of BALB/c mice. Furthermore, the HAP hydrogels showed better anti-wrinkle effect in wrinkled mice model that was further confirmed by the higher collagen deposition. The HA hydrogels prepared in this study found to be mechanically stable, biocompatible and injectable that could be used as stable fillers for diverse applications, including cell therapy and tissue engineering applications.

## CRediT authorship contribution statement

**Mohanapriya Murugesan:** Methodology, Formal analysis, Conceptualization. **Ramya Mathiyalagan:** Methodology, Formal analysis, Conceptualization. **Zelika Mega Ramadhania:** Methodology, Formal analysis. **Jinnatun Nahar:** Methodology, Formal analysis. **Cuong Hung Luu:** Validation, Software. **V.H. Giang Phan:** Validation, Software. **Deok Chun Yang:** Resources, Investigation, Conceptualization. **Qihui Zhou:** Validation, Software. **Se Chan Kang:** Resources, Formal analysis. **Thavasyappan Thambi:** Supervision, Funding acquisition, Formal analysis, Conceptualization.

## Data availability statement

The data presented in this study are available on request from the corresponding author.

## Ethics approval and consent to participate

All the animal experiments were performed in accordance with the guidelines of the Institutional Animal Experiment Committee at Kyung Hee University. The Institutional Committees of Kyung Hee University approved our experiments (Approval number: KHU-2022–0216).

## Declaration of competing interest

The authors declare no conflict of interest.
