# Supplementary material for: Tailoring hyaluronic acid hydrogels: Impact of cross-linker length and density on skin rejuvenation as injectable dermal fillers and their potential effects on the MAPK signaling pathway suppression
Source: Bioact Mater. 2025 Mar 8;49:154–71. doi: 10.1016/j.bioactmat.2025.03.002 (PMC11930439; doi:10.1016/j.bioactmat.2025.03.002)
Supplement: Multimedia component 1 [file mmc1.docx]

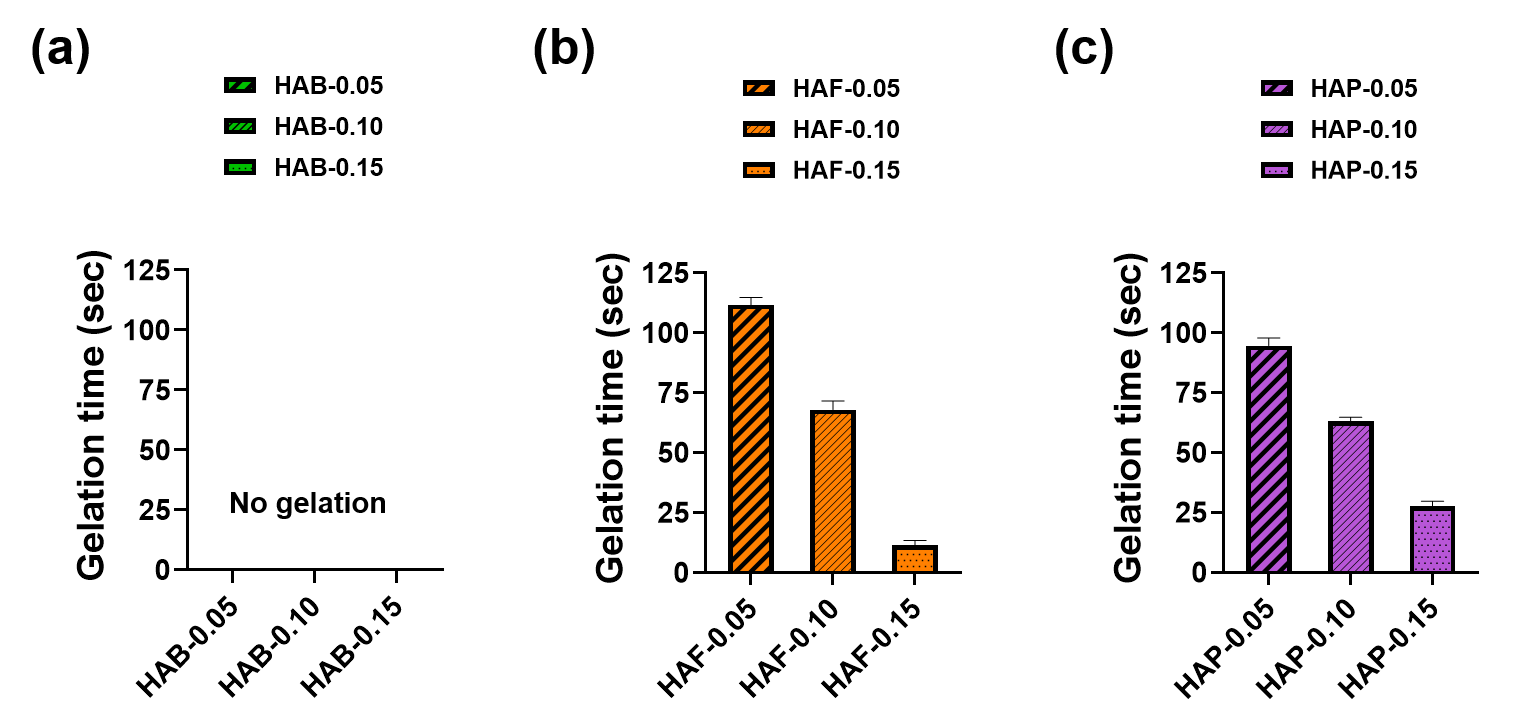


**Fig. S1.** The gelation time of various HA hydrogels with increasing concentrations of cross-linkers. Error bars in the graph represent the mean ± SD (n = 3).


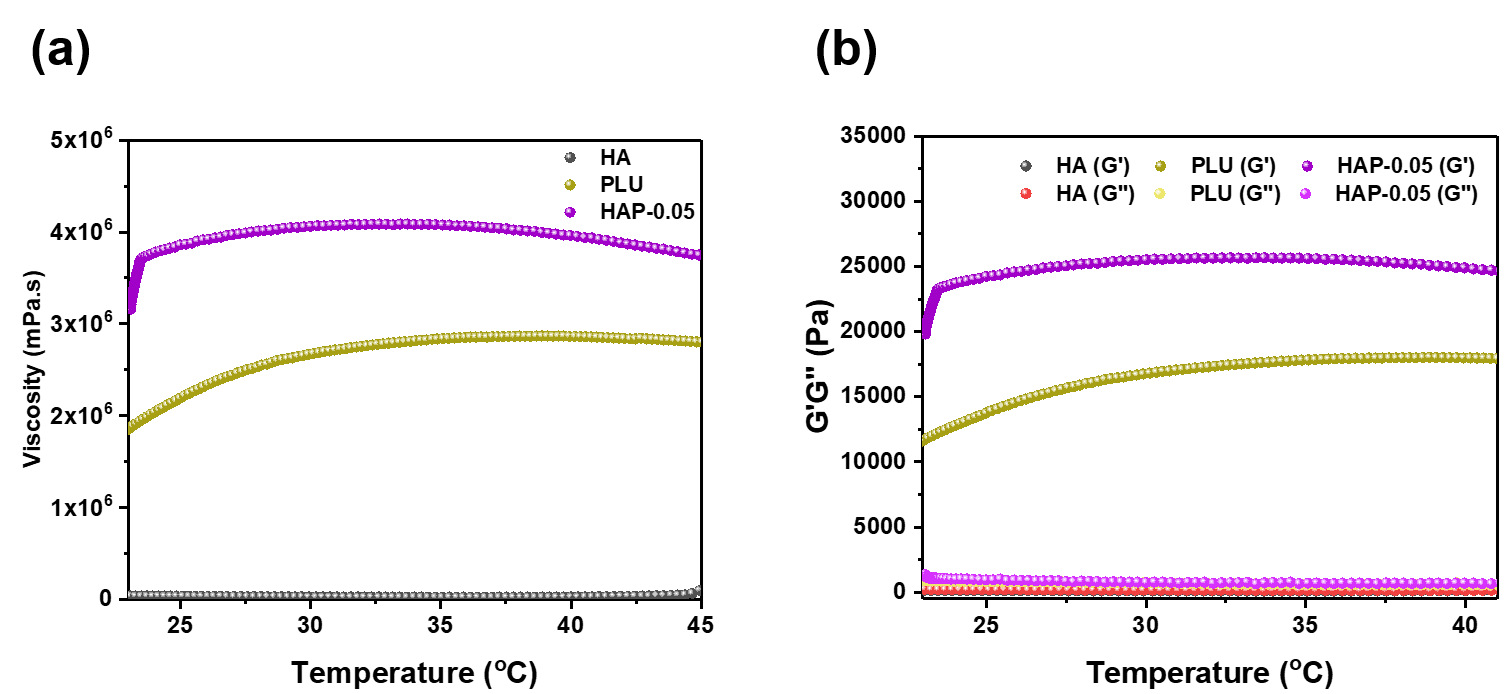


**Fig. S2.** Rheological properties of hydrogels. (a) Complex viscosity curves of PLU and HAP-0.05 hydrogels as a function of temperature. (b) Change in storage modulus (G’) and loss modulus (G’’) of the PLU and HAP-0.05 hydrogels as a function of temperature.


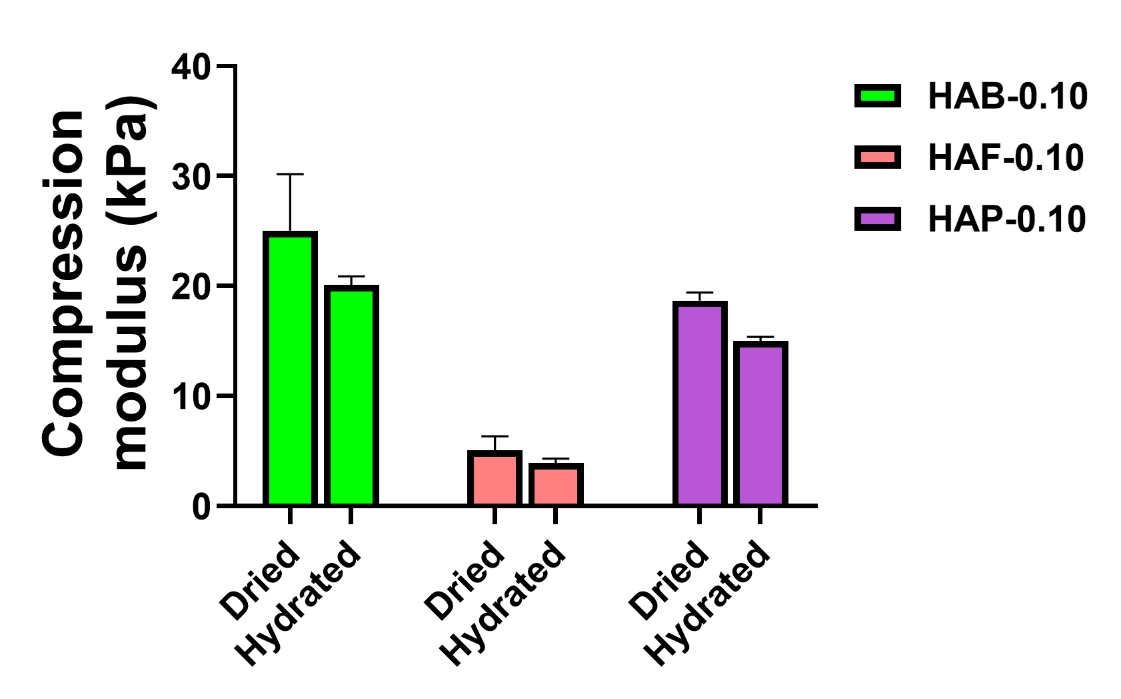


**Fig. S3**: The compression modulus of different HA-based hydrogels in dried and hydrated conditions.


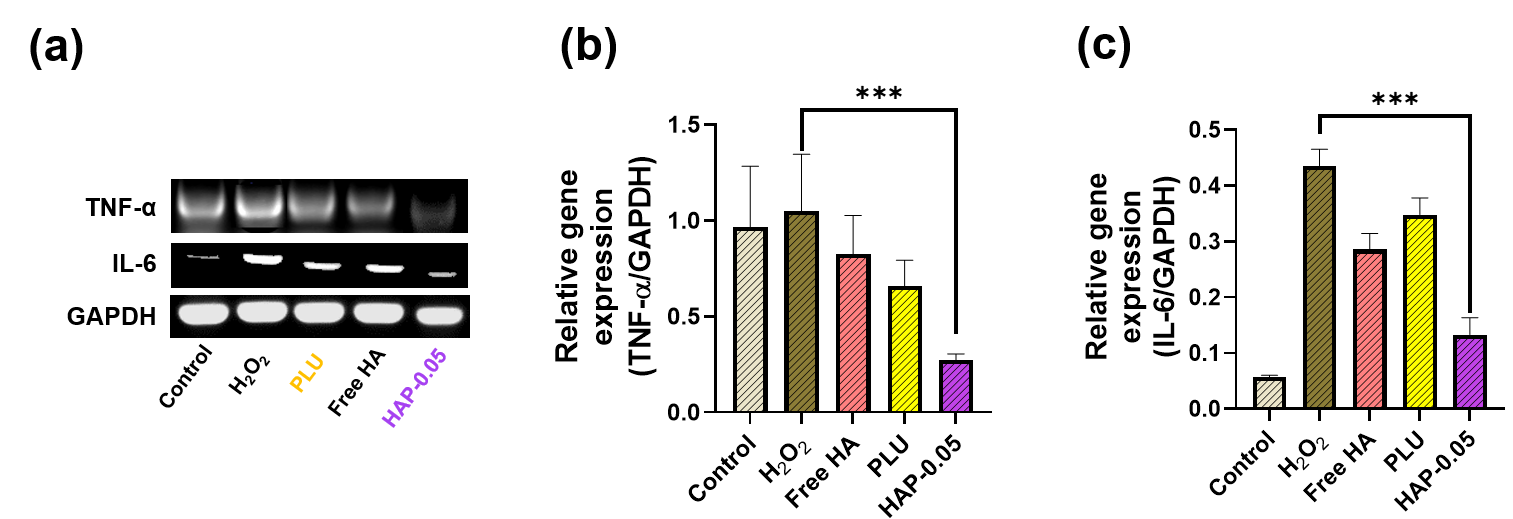


**Fig. S4**: (a) RT-PCR results showing IL-6 and TNF-α expression in HaCaT cells. (b) and (c) represent the corresponding quantitative analysis of bands using ImageJ analysis. Asterisks (*) denote statistically significant differences calculated using a one-way ANOVA test with Tukey’s post hoc test (p < 0.001).


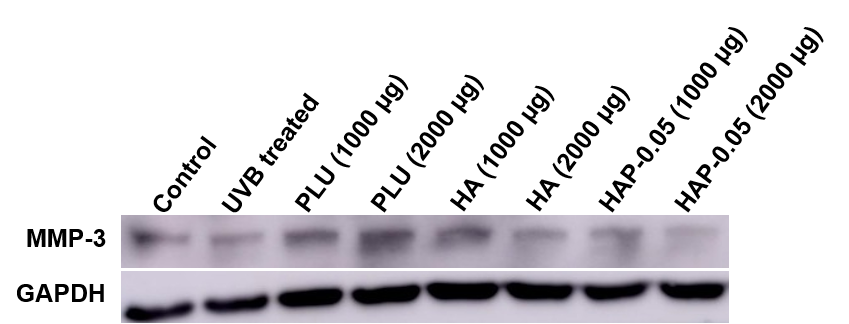


**Fig. S5.** Western blot analysis of MMP-3 protein expression in HDF cells treated with HA, PLU, and HAP hydrogels at two different concentrations (1000 µg and 2000 µg), following UVB irradiation. The bands represent MMP-3 expression levels, with HAP hydrogel treatment effectively downregulating MMP-3 expression in a concentration-dependent manner. In contrast, PLU and free HA treatments showed no suppression of MMP-3 expression, as their band intensities were similar to those of the untreated control groups.


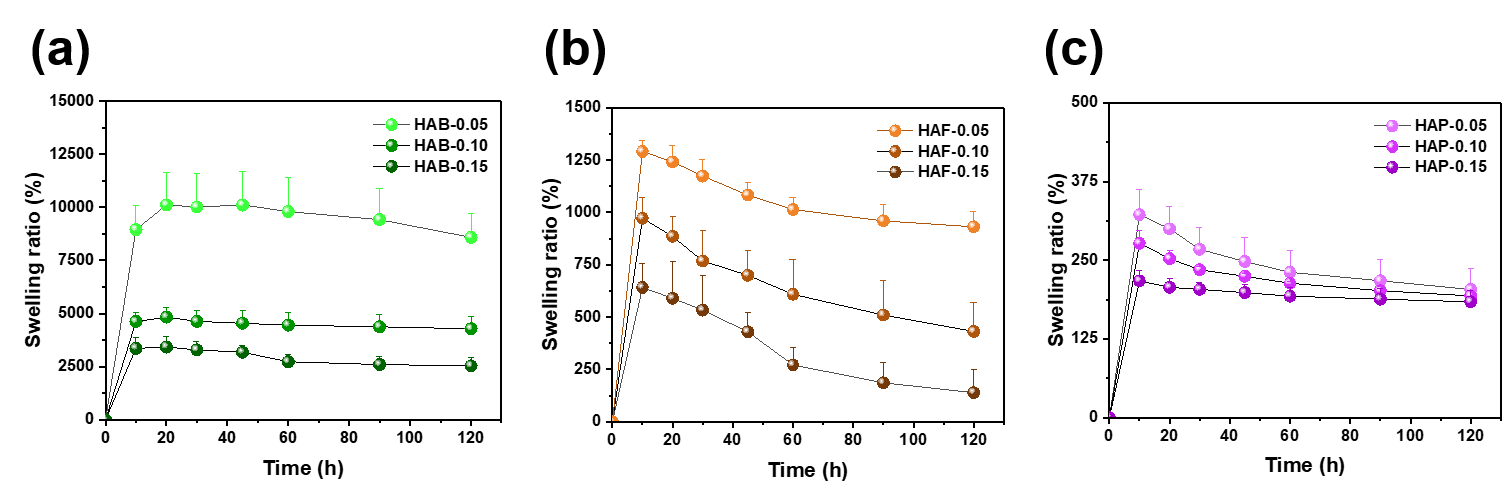


**Fig. S6.** Swelling ratio of hydrogels in physiological conditions (37 °C, pH 7.4). Error bars in the graph represent the mean ± SD (n = 3).


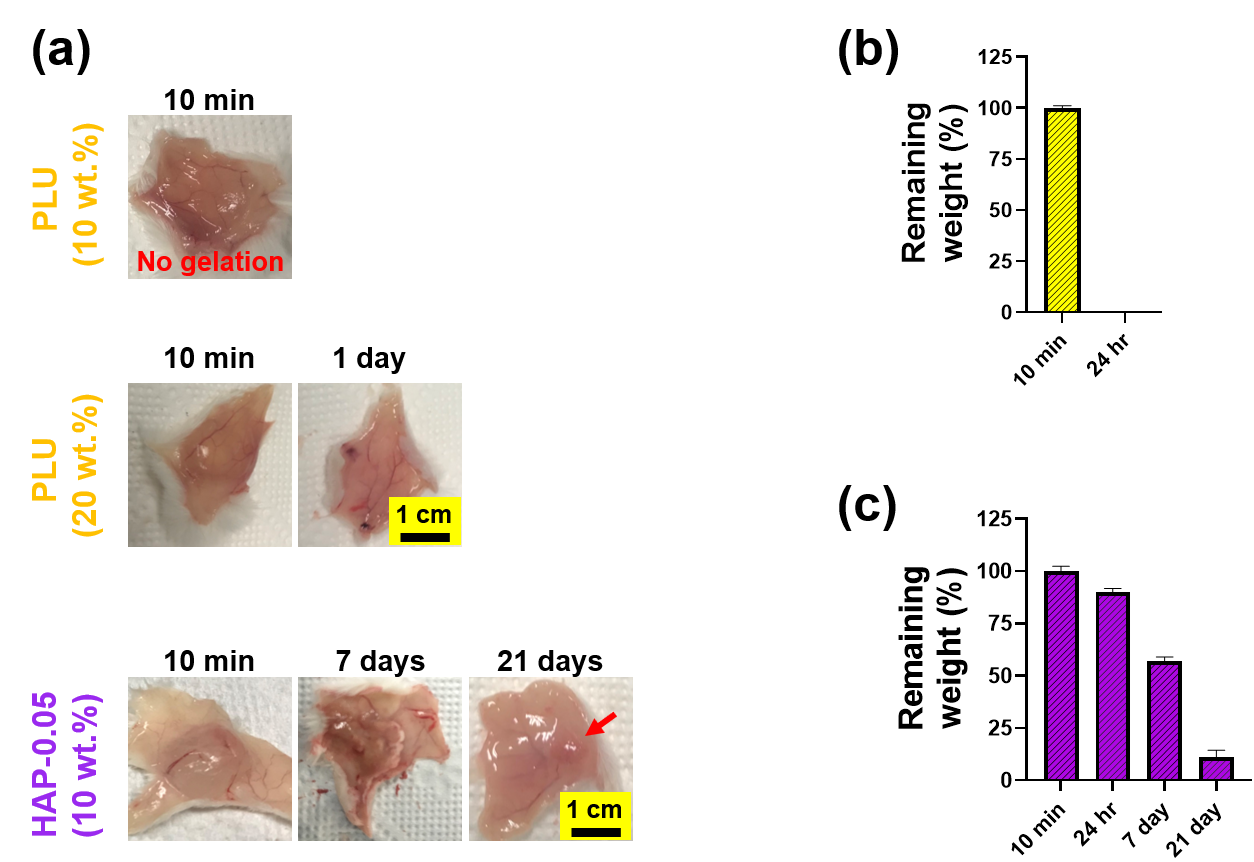


**Fig. S7.** (a) Representative photographic images depict the remaining hydrogels at various time points in mice following the injection of different hydrogel formulations. The red arrow highlights the remaining gels. Changes in the weight of the remaining (b) PLU hydrogel and (c) HAP-0.05 hydrogel over time are presented. To examine in vivo gel formation, 200 µL of hydrogel precursors are subcutaneously injected into the backs of BALB/c mice. The values represent the mean ± SD (n = 3).

**
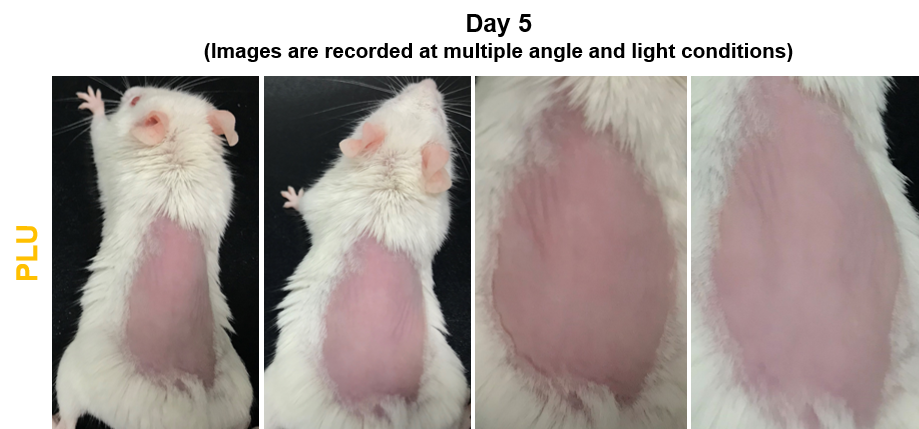
**

**Fig. S8.** Skin surface images of the mice after PLU hydrogel treatment at day 5 and the images are recorded at multiple angle and light conditions.


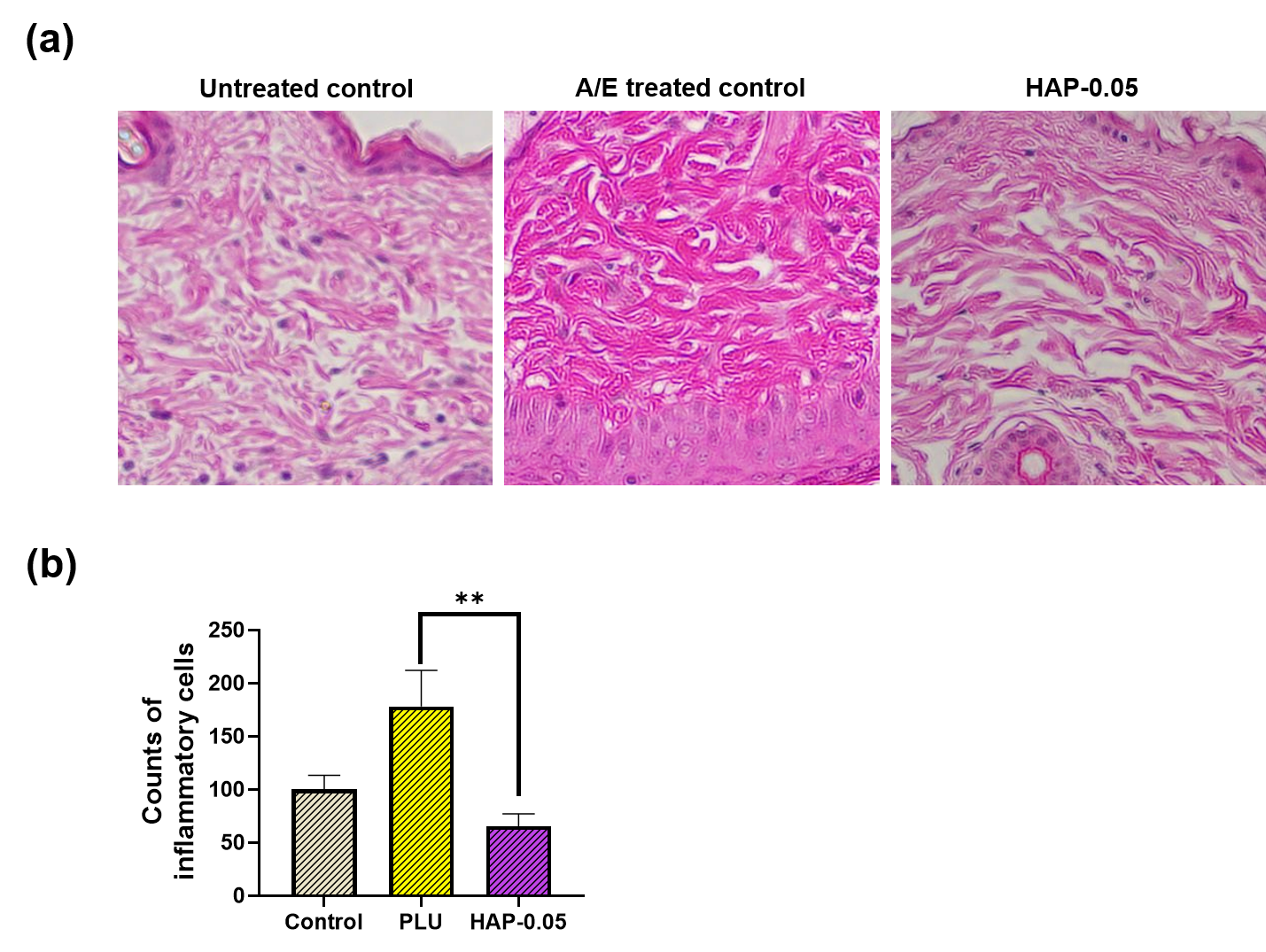


**Fig. S9**. (a) Histological images of the implantation site showing minimal immune response in hydrogel-implanted mice. (b) Quantification of inflammatory cells in each group. Statistically significant differences were determined using a one-way ANOVA test with Tukey’s post hoc test (p < 0.005), as indicated by asterisks (*).

**Table S1.** Reaction conditions, degree of substitution (DS), and number-average molecular weight of three different hydrogels with different cross-linking density.

| **Sample name** | **HA molecular weight (g/mol)^a^** | **Feed ratio of cross-linker^b^** | **DS of cross-linker^c^** | **Molecular weight of cross-linked HA (g/mol)** |
| --- | --- | --- | --- | --- |
| **HAB-0.05** | 200,000 | 0.05 | 4.0±0.07 | 204,658 |
| **HAB-0.10** | 200,000 | 0.10 | 6.55±0.28 | 206,968 |
| **HAB-0.15** | 200,000 | 0.15 | 12.14±0.33 | 212,915 |
|  | | | | |
| **HAF-0.05** | 200,000 | 0.05 | 3.75±0.27 | 207,660 |
| **HAF-0.10** | 200,000 | 0.10 | 5.97±0.18 | 212196 |
| **HAF-0.15** | 200,000 | 0.15 | 9.20±0.35 | 218794 |
|  | | | | |
| **HAP-0.05** | 200,000 | 0.05 | 1.98±0.09 | 331,226 |
| **HAP-0.10** | 200,000 | 0.10 | 4.15±0.15 | 475,045 |
| **HAP-0.15** | 200,000 | 0.15 | 5.13±0.23 | 539,996 |

^a^Provided by the Lifecore Biomedical Lifecore Biomedical, Chaska, MN

^b^Feed molar ratio of BDDE, FA or PLU to the HA repeating unit

^c^Degree of substitution was calculated using the ^1^H NMR spectra.

**Table S2.** Injectability rating of 10 wt.% of HAB, HAF, and HAP hydrogels.

| **Sample name** | **Needle size (Gauge)** | | | |
| --- | --- | --- | --- | --- |
|  | **21G** | **22G** | **24G** | **26G** |
| **HAB-0.05** | ********* | ******** | ******* | ******* |
| **HAB-0.10** | ******** | ******* | ******* | ****** |
| **HAB-0.15** | ******** | ****** | ****** | ***** |
|  | | | | |
| **HAF-0.05** | ********* | ******** | ******* | ****** |
| **HAF-0.10** | ******** | ******* | ****** | ***** |
| **HAF-0.15** | ******* | ******* | ****** | ***** |
|  | | | | |
| **HAP-0.05** | ********* | ******** | ******* | ******* |
| **HAP-0.10** | ********* | ******** | ******** | ******* |
| **HAP-0.15** | ********* | ******** | ******* | ****** |

***** a perfect flow

**** flow with slight pressure

*** flow with high pressure

** flow with very high pressure

* no flow

**Table S3.** Calculation of parameters in the Flory–Rehner equation.

|  | **HAB-0.05** | **HAB-0.10** | **HAB-0.15** | **HAF-0.05** | **HAF-0.10** | **HAF-0.15** | **HAP-0.05** | **HAP-0.10** | **HAP-0.15** |
| --- | --- | --- | --- | --- | --- | --- | --- | --- | --- |
| ***V*_1_ (mL/mol)** | 18.01 | 18.01 | 18.01 | 18.01 | 18.01 | 18.01 | 18.01 | 18.01 | 18.01 |
| ***ρ*_1_ (g/mL)** | 0.997 | 0.997 | 0.997 | 0.997 | 0.997 | 0.997 | 0.997 | 0.997 | 0.997 |
| ***ρ*_2_ (g/mL)** | 1.8 | 1.8 | 1.8 | 1.8 | 1.8 | 1.8 | 1.8 | 1.8 | 1.8 |
| ***V*_2_** | 0.005 | 0.011 | 0.016 | 0.041 | 0.054 | 0.079 | 0.147 | 0.167 | 0.203 |
| ***χ*** | 0.502 | 0.504 | 0.505 | 0.514 | 0.518 | 0.526 | 0.549 | 0.556 | 0.568 |
| ***ν*_e_ (nmol/mL)** | 0.1 | 1.1 | 3.7 | 126.5 | 349.1 | 1515.3 | 16013.1 | 26524.5 | 58051.0 |

Calculation of crosslinking density

To calculate the crosslink density of hydrogel systems based on their relationship with the swelling ratio, we employed the Flory–Rehner equation, presented below as Equation (1). The swelling ratio tests were conducted using PBS, chosen as a favorable solvent for HA-based hydrogels.

|  | $\ln\left( 1-V_{2} \right)+V_{2}+\chi V_{2}^{2}=V_{1}\upsilon_{e}\left( \frac{V_{2}}{2}-V_{2}^{\frac{1}{3}} \right)$ | (1) |
| --- | --- | --- |

Here, *V*_1_ represents the molar volume of PBS, *χ* denotes the Flory–Huggins interaction parameter for the HA hydrogel network in PBS, and *V*_2_ is the volume fraction of the polymer in its swollen state.

*V*_2_ is given by Equation (2):

|  | $V_{2}=\left[ 1+\left( \frac{W_{\mathrm{eq}}-W_{0}}{W_{0}} \right)\left( \frac{\rho_{2}}{\rho_{1}} \right) \right]^{-1}$ | (2) |
| --- | --- | --- |

*W*_0_ is the mass of the HA hydrogels prior to the swelling test, while *W*_eq_ corresponds to the point where the measured *W* over time reaches stability, signifying equilibrium. *ρ*_1_ and *ρ*_2_ are the mass densities of PBS and HA, respectively. Given the minimal difference in mass density compared to water, we used an approximate value for *ρ*_1_ of 0.997 g/mL. The experimentally determined value of *ρ*_2_ was 1.8 g/mL.

Assuming the *χ* value exceeds 0.5, χ can be approximated through *V*_2_ using Equation (3).

|  | $\chi=\frac{1}{2}+\frac{V_{2}}{3}$ | (3) |
| --- | --- | --- |

Based on the parameters outlined above, *ν*_e_ was calculated using Equation (4) below. The resulting value indicates the number of network chains per unit volume that contribute to the polymer’s elastic properties when in the swollen state.

|  | $\upsilon_{e}=\frac{\ln\left( 1-V_{2} \right)+V_{2}+\chi V_{2}^{2}}{V_{1}\left( \frac{V_{2}}{2}-V_{2}^{\frac{1}{3}} \right)}$ | (4) |
| --- | --- | --- |
